# On-patient medical record and mRNA therapeutics using intradermal microneedles

**DOI:** 10.1038/s41563-024-02115-4

**Published:** 2025-02-24

**Authors:** Jooli Han, Maria Kanelli, Yang Liu, John L. Daristotle, Apurva Pardeshi, Timothy A. Forster, Ari Karchin, Brandon Folk, Lukas Murmann, Lisa H. Tostanoski, Sebastian E. Carrasco, Shahad K. Alsaiari, Erika Yan Wang, Khanh Tran, Linzixuan Zhang, Behnaz Eshaghi, Lauren Levy, Sydney Pyon, Charles Sloane, Stacey Qiaohui Lin, Alicia Lau, Collin F. Perkinson, Moungi G. Bawendi, Dan H. Barouch, Frédo Durand, Robert Langer, Ana Jaklenec

**Affiliations:** 1 Koch Institute for Integrative Cancer Research, Massachusetts Institute of Technology, Cambridge, MA USA; 2 Department of Biomedical Engineering, Seoul National University College of Medicine, Seoul, Republic of Korea; 3 Computer Science and Artificial Intelligence Laboratory, Massachusetts Institute of Technology, Cambridge, MA USA; 4 Global Health Labs, Bellevue, WA USA; 5 Center for Virology and Vaccine Research, Beth Israel Deaconess Medical Center, Harvard Medical School, Boston, MA USA; 6 Laboratory of Comparative Pathology, Weill Cornell Medicine, Memorial Sloan Kettering Cancer Center, Rockefeller University, New York, NY USA; 7 Department of Chemical Engineering, Massachusetts Institute of Technology, Cambridge, MA USA; 8 Department of Chemistry, Massachusetts Institute of Technology, Cambridge, MA USA; 9 Harvard Medical School, Boston, MA USA; 10 The Ragon Institute of Mass General Brigham, MIT, and Harvard, Cambridge, MA USA

**Keywords:** Biomaterials - vaccines, Nanoparticles, Biomedical materials

## Abstract

Medical interventions often require timed series of doses, thus necessitating accurate medical record-keeping. In many global settings, these records are unreliable or unavailable at the point of care, leading to less effective treatments or disease prevention. Here we present an invisible-to-the-naked-eye on-patient medical record-keeping technology that accurately stores medical information in the patient skin as part of microneedles that are used for intradermal therapeutics. We optimize the microneedle design for both a reliable delivery of messenger RNA (mRNA) therapeutics and the near-infrared fluorescent microparticles that encode the on-patient medical record-keeping. Deep learning-based image processing enables encoding and decoding of the information with excellent temporal and spatial robustness. Long-term studies in a swine model demonstrate the safety, efficacy and reliability of this approach for the co-delivery of on-patient medical record-keeping and the mRNA vaccine encoding severe acute respiratory syndrome coronavirus 2 (SARS-CoV-2). This technology could help healthcare workers make informed decisions in circumstances where reliable record-keeping is unavailable, thus contributing to global healthcare equity.

## Abstract (web summary)

Intradermal microneedles for the co-delivery of mRNA and near-infrared fluorescent microparticles are used in combination with deep learning-based image analysis for the simultaneous administration of therapeutics and registry of patient information records into the skin.

## Main

Medical interventions often require specifically timed doses, necessitating accurate medical record-keeping. In particular, mRNA-based delivery systems have proved to be a versatile platform for vaccine and therapeutics development against incurable diseases^1–5^, often requiring multiple doses. To name a few examples, clinical trials for RNAactive CV7201 for rabies (CureVac)^6^, RNActive CV9103 for prostate cancer (CureVac)^7^ and mRNA-4157-P201 for melanoma (Moderna)^8^ required 3, 5 and 9 dose administrations, respectively. Many other therapeutics and most of the recommended childhood immunizations require multiple doses (for example, the hepatitis B and polio vaccines) as well^9–11^. Failure to complete these timed doses risks suboptimal protection. Currently, as many as 40% of patients fail to comply with medical treatments around the globe, and poor adherence is responsible for 125,000 annual deaths in the United States alone^12,13^. In sub-Saharan Africa, 35% of children between 12 and 23 months fail to complete recommended childhood vaccinations^14,15^. Several factors contribute to these gaps, including lack of access or inability to afford medical treatments and vaccines, but a major contributor is inadequate medical record-keeping systems^16,17^. Conventional methods like paper-based cards and online databases present serious risks of losing access to medical histories, and therefore the risk of missing or mistimed follow-up doses. For that reason, a handful of medical record-keeping means have emerged based on fingerprint scanning^18^, cell phone applications^19–21^, microchips^22^ and more. However, these approaches have raised privacy concerns related to individual, identifiable medical data storage in a centralized database, which poses a risk of data breech, misuse or quality implications^23–27^.

**Fig. 1:**
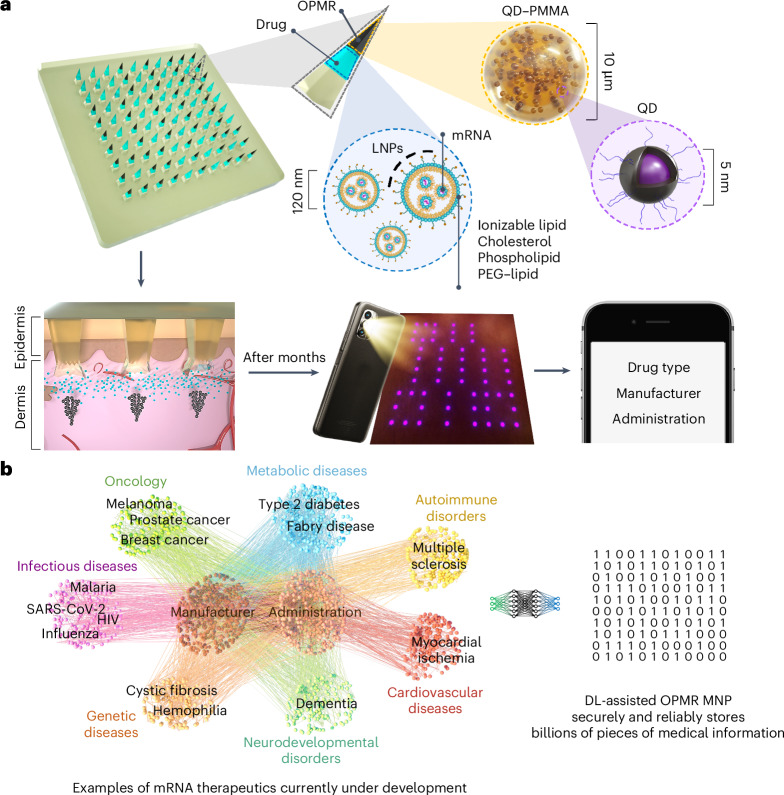
Schematic of the OPMR technology used for medical information record-keeping. **a**, NIR fluorescent QD dye encapsulated in PMMA microparticles (black component of the needle tip) is co-loaded with mRNA encapsulated in LNPs (light blue component of the needle tip) into microneedles that are held intact by a dissolvable polymer backing. Upon MNP application, dye microparticles are deposited into the dermis layer in a predefined pattern that encodes medical information, while mRNA-LNPs are uptaken by immune cells, inducing immunogenicity. NIR patterns are imaged and processed for medical information retrieval on a screen. **b**, The deep learning (DL)-assisted OPMR technology offers a large encoding capacity in the 10^6^ to 10^9^ range by leveraging the binary feature of OPMR microneedle bits, making the technology applicable to the fast-growing number of mRNA therapeutics currently under development.

**Extended Data Fig. 1:**
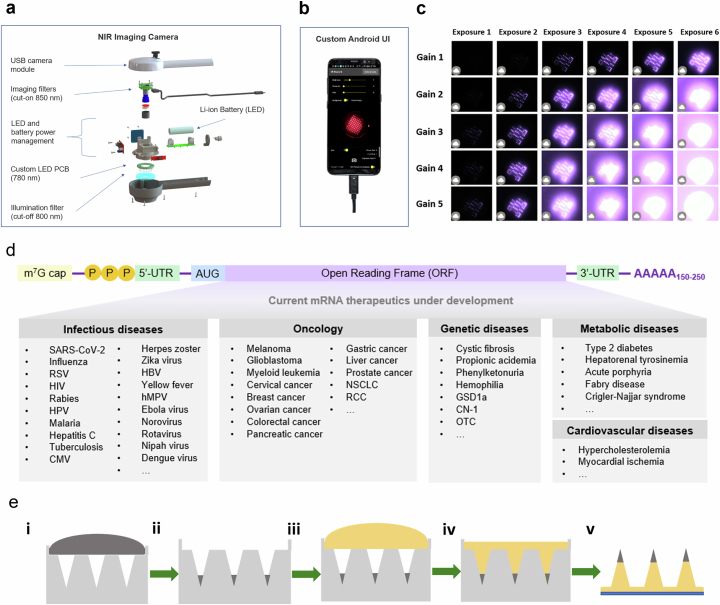
NIR QD microneedle-based on-patient medical recordkeeping (OPMR) system. **(a)** OPMR dye, once deposited in the skin, can be detected by a custom-made handheld NIR imaging system. USB-connected NIR fluorescence imaging system involving a custom LED module emits shorter-wavelength NIR light at 780 nm and a USB-connected camera module that captures the excited QD fluorescence image at longer-wavelength NIR light at >850 nm. **(b)** Android smartphone with a custom-developed phone application ‘IR Record’ is optimized to capture the OPMR NIR dye signal and saves images. **(c)** The software takes 30 consecutive images with six different exposure and five different gain settings. This bracket scanning method allows capturing of NIR signals with varying intensities over time. Among the 30 images, one image with the best reading results gets automatically chosen and processed. **(d)** This OPMR system can be co-loaded with mRNA therapeutics for cancer, infectious, genetic, metabolic, cardiovascular, and neurodevelopmental diseases, and more currently under development from around the globe. The open reading frame (ORF) region of mRNA strands makes the technology easily applicable for a variety of diseases. **(e)** Schematic of MNP fabrication process**. i**. QD-PMMA solution is dispensed on top of a PDMS negative mold, which is made with a custom-designed metal master mold. **ii**. QD-PMMA microparticles are concentrated at the needle tips either by centrifugation or application of vacuum beneath the PDMS mold. **iii**. The PVP-PVA polymer blend solution is dispensed on top of the PDMS mold to fill the rest of the needles. **iv**. The polymer solution enters the needle cavities via centrifugation or application of vacuum and form needles and a thin layer of backing for the MNP hold to microneedles intact. **v**. Once the polymer dries, a Delrin backing is attached on the patch and the patch is removed vertically from the PDMS mold.

We developed a robust on-patient medical record-keeping (OPMR) technology using a dissolvable microneedle patch (MNP) that delivers a quantum dot (QD)-based near-infrared (NIR) fluorescent dye encapsulated in poly(methyl methacrylate) (PMMA) microparticles into the skin to encode medical information^28^ (Fig. 1a). This dye, once deposited into the dermis, is invisible to the naked eye, offering patient data privacy and anonymity, but provides discrete NIR signals that can be detected using a NIR imaging system (Extended Data Fig. 1a–c). By depositing the dye in a predefined pattern that correlates to a specific set of information, the technology can be imaged by healthcare workers to support next-dose decisions without requiring internet connectivity or the use of centralized databases.

Herein, to enable the OPMR with excellent information capacity, security and reliability, we designed the MNP architecture and administration for consistent and optimal data transfer and longevity; achieved an information capacity of billions of encoded patterns using an error correcting code; and developed a temporally and spatially reliable information retrieval system using machine learning. Further, we successfully co-delivered the OPMR with a potent mRNA vaccine encapsulated in lipid nanoparticles (LNPs) that encodes the SARS-CoV-2 spike protein (Fig. 1a). This demonstrates that our OPMR–mRNA MNP technology can co-deliver mRNA therapeutics and corresponding medical information simultaneously. The scope of its application is potentially expandable to any mRNA therapeutics, considering its biocompatibility with mRNA-LNPs and its large encoding capacity in the range of 10^6^ to 10^9^ that accommodates the fast-growing number of mRNA therapeutics under development (Fig. 1b and Extended Data Fig. 1d). This tool could help healthcare workers make informed decisions on follow-up doses in the field where reliable record-keeping is unavailable, and therefore improve medical adherence and complete immunization for the global population.

## OPMR MNP materials and architecture

**Fig. 2:**
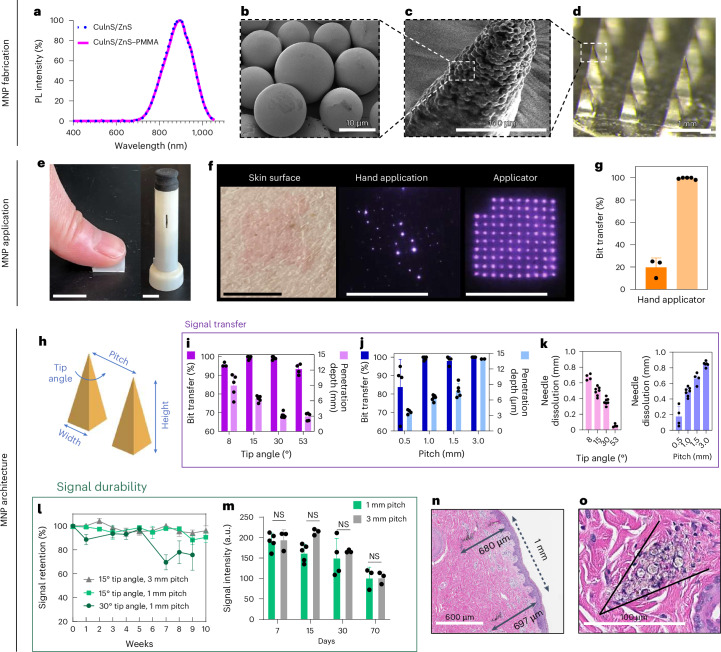
MNP materials, design and application for effective OPMR delivery. **a**, Normalized photoluminescence (PL) intensity of CuInS_2_/ZnS QD that peaks at 897 nm with or without PMMA encapsulation. **b**, SEM image of PMMA microparticles with QD nanocrystals encapsulated within. (SEM repeated twice.) **c**, SEM image of a single microneedle tip loaded with QD–PMMA microparticles. (SEM performed once.) **d**, Optical image of a MNP loaded with QD–PMMA microparticles at the needle tips. (Optical imaging repeated >30 times.) **e**, Photo of MNP applications by hand and with a spring applicator. Scale bars, 1 cm. **f**, The OPMR MNPs do not leave visible footprints; better NIR bit transfer is exhibited with an applicator, as shown in the photos. Scale bars, 1 cm. **g**, Data showing that better NIR bit transfer is achieved with an applicator; *n* ≥ 3. It was done with biological replicates of 3–5 animals. **h**, MNP architecture variables that can affect OPMR quality. **i**, Bit transfer and skin penetration depth evaluated for different needle tip angles; *n* ≥ 4, biological, s.d. **j**, Bit transfer and skin penetration depth evaluated for different pitches; *n* ≥ 4, biological, s.d. **k**, Needle dissolution evaluated for different tip angles and pitches; *n* ≥ 4, biological, s.d. (Experiments in **e**–**k** were performed in ex vivo pig skin.) **l**, Signal retention of different MNP architectures over ten weeks; *n* ≥ 4, biological, s.d. **m**, Signal intensities of applied MNPs with 1 mm and 3 mm pitches for 70 days; *n* ≥ 3, biological, s.d.; NS, not significant. **n**,**o**, Representative histology image of two bits (**n**; histology imaging repeated >30 times) and of spherical QD–PMMA microparticles (**o**) deposited well within the dermis of pig skin. (Experiments in **l**–**o** were performed in vivo in Yorkshire pigs). **o**, The black arrow in the figure highlights where a microneedle tip was inserted and left a trace of quantum dot microparticles.

The NIR signal encoding was generated from CuInS_2_/ZnS QDs with a photoluminescence quantum yield of 77% and photoluminescence intensity peak at 890–897 nm (ref. ^28^; Fig. 2a). The QDs were encapsulated in PMMA microparticles to increase the size of the particles and thus mitigate biological clearance and enhance the stability and biocompatibility of the system^28–31^. The PMMA encapsulation did not result in a shift of the peak emission wavelength (Fig. 2a) or major decrease of the photoluminescence quantum yield, as it remained at 73% post-encapsulation. The diameter of the QD–PMMA microparticles (OPMR dye) was tuned to be roughly 10 μm, and the average size was confirmed with scanning electron microscopy (SEM; Fig. 2b). For the evaluation of effective OPMR delivery, MNPs with a 10 × 10 array were used, and were loaded with OPMR dye at the needle tips and a polymer blend as backing (Extended Data Fig. 1e). Each of the 100 microneedles contains OPMR dye at the tip (Fig. 2c), and collectively they form a 10 × 10 array, with each needle corresponding to a single NIR bit (Fig. 2d). Because accurate intradermal information delivery is a critical first step, the MNP application and architecture were designed for consistent dye transfer and optimal signal durability. Towards that end, the three most critical parameters were investigated: (1) bit transfer, the percentage of NIR-labelled needles transferred to and detectable in the skin as bits out of the total number of applied dye-loaded microneedles, as this indicates the initial information encoding to the skin; (2) penetration depth, the intradermal depth where the needle tips deposit the dye, because this may affect signal durability; and (3) needle dissolution, as this translates to the amount of cargo delivered to the skin. For these studies, four needles in one corner of a 10 × 10 MNP were removed for patch orientation, leaving the patch with 96 needles.

**Extended Data Fig. 2:**
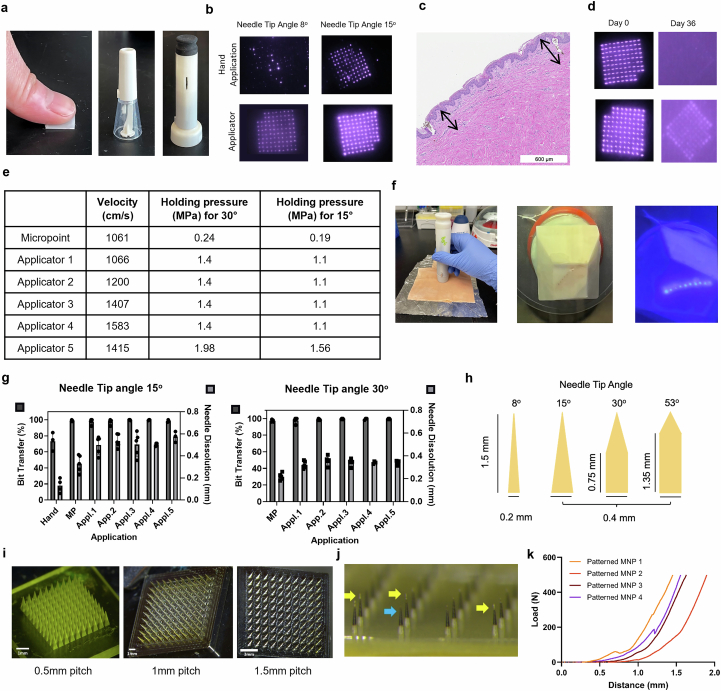
Evaluation of MNP application with different applicators and MNP architectures. **(a)** MNPs were applied to *ex vivo* pig skin by hand (left), commercial (Micropoint, Shenzhen, China) (middle) and custom spring-loaded applicators (right). **(b)** NIR images of MNPs with two different needle tip angles show that bits are better transferred when applied with an applicator than by hand. **(c)** Histological imaging of pig skin where MNPs did not penetrate more than 250 μm in depth, with hand application, leaving most of the dyes deposited near the epidermis layer, Imaging was performed >30 times. **(d)** *In vivo* NIR images of shallowly applied MNPs on day 0 and day 36 post-application in pigs demonstrated a dramatic decrease after 1 month, leading to the assumption that they are shed off with the top layer of the epidermis and that a deeper deposition of the dye can lead to a longer NIR signal durability. **(e)** The Micropoint and five custom-designed spring-loaded applicators with tunable impact velocities and holding pressures were tested for MNPs with two different tip angles. **(f)** Applicators were tested on pig skin (left). After the application, tissue was fixed in formalin and embedded in paraffin for cross-sectional evaluation of the maximum needle penetration and dye deposition depths (middle). Furthermore, parts of the skin tissue were frozen and fixed in Optimal Cutting Temperature compound for cross-sectional imaging to detect the presence of the NIR bits in the dermis, showing penetration of a 10-needle array (right). **(g)** NIR bit transfer and needle dissolution results for 2 different needle tip angles and for different application parameters (needles have 1.5 mm height, 0.4 mm base and 1 mm pitch), n = >4, biological, S.D. **(h)** Four different microneedle tip angles were tested for MNP architecture optimization, n = >5, biological. **(i)** Four different pitches, 0.5 mm, 1 mm, 1.5 mm, and 3 mm, were tested for MNP architecture optimization. **(j)** For needles with 8° tip angle, the dye does not reach the very ends of the needle tips (pointed out with yellow arrows), and the needles are more prone to breakage upon removal from the PDMS negative mold because of their thin structures at the tips (pointed out with blue arrows). **(k)** To assess the mechanical robustness of the MNP upon skin penetration, we performed mechanical compression tests on 10×10 patterned MNPs (n = 5) using Instron 5943 (Norwood, MA). Microneedles must pierce the stratum corneum without rupturing or bending for proper skin penetration (https://link.springer.com/article/10.1007/s40820-021-00611-9). The pressure required to puncture human skin is known to be roughly 100 psi, which is equivalent to 0.689 MPa (https://pubmed.ncbi.nlm.nih.gov/1757138/). Therefore, the minimum force required to puncture human skin with our patterned MNP (roughly 50 microneedles with the needle base dimension of 400 µm x 400 µm) is 5.512 N (**Eq. 1**), which means one microneedle patch needs to endure minimum of 5.512 N of compression force to pierce human skin. F_min_ = P x A_max_ = (6.89 × 10^5^ N/m^2^) * (8 *10^−6^ m^2^) = 5.512 N (Eq. 1). With Instron 5943, the microneedle patches were compressed at a rate of 5 mm/min, and the maximum load, load at break, and Young’s modulus were measured with Instron static load cell (±500 N) and Instron Bluehill 3 software. For all patches, the compression force measurements reached the upper limit of the load cell (500 N) before the platens reached the base of the needles, indicating that our microneedle patch can endure more than 500 N, easily exceeding the minimum force to endure for human skin penetration.

First, we compared the results of applying MNPs by hand and with spring applicators (Fig. 2e and Extended Data Fig. 2a). The hand application resulted in poor and inconsistent dye transfer when inspected with the NIR imaging system (Fig. 2f), with less than 20% bit transfer, while applicators exhibited 100% bit transfer (*n* = 3–5; Fig. 2g and Extended Data Fig. 2b). For penetration depth, needle tips did not reach deeper than 250 μm with the hand application, leaving most of the dye deposited near the epidermis (Extended Data Fig. 2c) and resulting in most of the NIR signals being gone after one month (Extended Data Fig. 2d). This led to an assumption that a deeper dye deposition with a proper applicator is necessary for durable NIR signals. Towards that end, custom spring applicators with a range of impact velocities and holding pressures (Extended Data Fig. 2e) were tested in ex vivo pig skin (Extended Data Fig. 2f), leading to the selection of an impact velocity of 1,407 cm s^–1^ and holding pressure of 1.1 MPa for use in the rest of the studies (Extended Data Fig. 2g).

Second, we optimized two MNP design variables that affected the MNP performance: (1) needle tip angle, which correlates to the needle width-to-height ratio and sharpness of the microneedle, and (2) pitch, which is the spacing between two needles from centre to centre (Fig. 2h). Four different tip angles, 8°, 15°, 30° and 53° with a fixed 1.5 mm needle height (Extended Data Fig. 2h), and four different pitches, 0.5 mm, 1 mm, 1.5 mm and 3 mm with a fixed 15° tip angle (Extended Data Fig. 2i), were tested for the maximum bit transfer, penetration depth and needle dissolution while maintaining the minimum patch size. For all four tip angles, penetration depths were well within the dermis layer^32^, but only the 15° and 30° tip angles resulted in 100% bit transfer (*n* = 4–7; Fig. 2i). The 8° tip angle was too fragile (Extended Data Fig. 2j) whereas the 53° tip angle was too blunt upon penetration. For needle spacings, all pitches deposited the dye within the dermis, but only the MNPs with a pitch equal to or larger than 1 mm resulted in 100% bit transfer (*n* = 4–7; Fig. 2j). For needle dissolution, a decreasing trend was observed with increasing tip angles, and an increasing trend was observed with increasing pitches (*n* = 4–7; Fig. 2k).

## OPMR MNP applications for effective delivery

**Extended Data Fig. 3:**
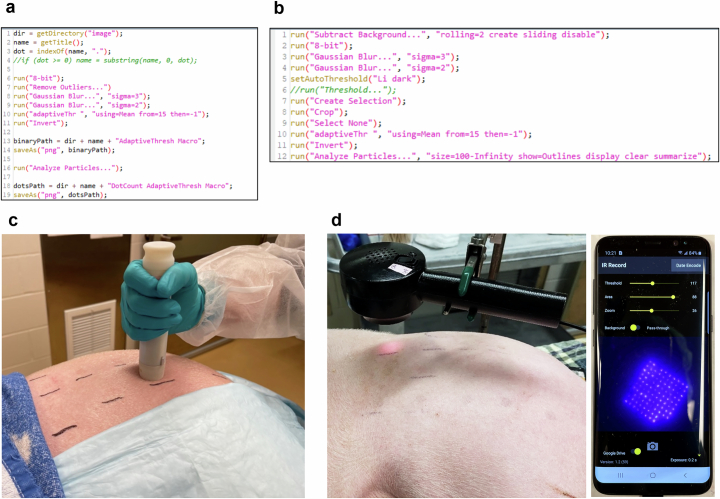
Adaptive threshold for NIR signal analysis. **(a)** The number of NIR bits were counted using ImageJ (National Institutes of Health, USA) and adaptive threshold method for NIR signal retention analysis. The results were used for MNP architecture optimization. **(b)** The brightness of NIR bits were computed using ImageJ and adaptive threshold method for NIR signal intensity analysis. The results were used for MNP architecture optimization. **(c)** Application of an MNP on swine using a custom applicator. **(d)** Applied MNP footprint was imaged with a custom NIR imaging system and analyzed with adaptive threshold algorithms.

Once the dye particles are deposited in the skin, the NIR signals must last to allow accurate information readings. For this purpose, the tip angles (15° and 30°) and pitches (1 mm and 3 mm) that resulted in 100% bit transfer were tested for signal durability in vivo. Signal durability involves two parameters: (1) signal retention, the percentage of detectable NIR bits out of the total transferred bits, and (2) signal intensity, the pixel brightness value (in a.u.) of NIR bits. Signal retention and signal intensity are key quantitative and qualitative indications of signal durability, respectively. For this study, a Yorkshire pig model was chosen due to its similar epidermis–dermis skin structure and mechanical properties compared to human skin^33^ (Extended Data Fig. 2k). Adaptive threshold algorithms were used for the signal retention (Extended Data Fig. 3a) and signal intensity (Extended Data Fig. 3b) analyses^34^. When three MNP groups (15°–1 mm, 15°–3 mm and 30°–1 mm) were applied (Extended Data Fig. 3c) and imaged weekly for 10 weeks (Extended Data Fig. 3d), the 15° groups resulted in 90.5 ± 9.04% signal retention on week 10, whereas the 30° group exhibited an inferior signal retention of 75.67 ± 22.12% on week 9 (*n* = 4–6; Fig. 2l). Considering that the 15°–1 mm and 15°–3 mm groups penetrated the skin more deeply (640 μm and 1,350 μm, respectively) than the 30°–1 mm group (300 μm; Fig. 2i,j), penetration depth seems to affect intradermal signal retention. When signal intensities of the two best performing groups (15°–1 mm and 15°–3 mm) were analysed for 70 days, although the overall intensity gradually decreased over time, no substantial difference occurred between the two groups (*n* = 3–5; Fig. 2m), indicating that signal intensity is not affected as long as dye is deposited deeper than a threshold depth (for example, 600 μm). Because 1-mm-pitch MNPs performed just as well as 3-mm-pitch MNPs while offering a nine times smaller patch size, a 10 × 10 MNP design with 1 mm pitch and 15° tip angle (0.4 mm × 0.4 mm × 1.5 mm) was selected for the rest of the studies. As a result, these optimized application and architecture parameters yielded a consistent penetration depth near 700 μm (Fig. 2n), effectively depositing the dye particles well within the dermis^32^ (Fig. 2o).

## Error correcting code for temporally robust encoding

**Extended Data Fig. 4:**
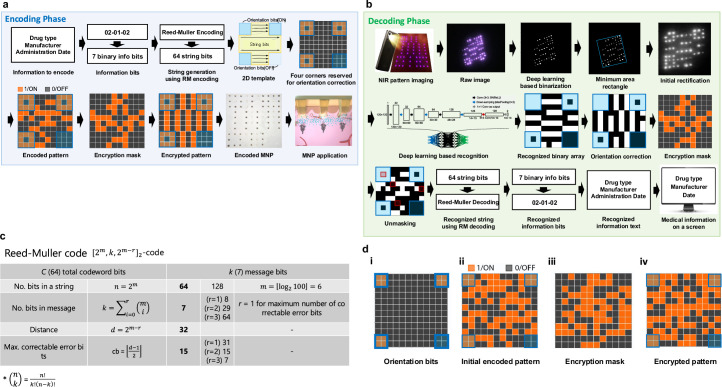
Encoding and decoding of medical information on MNP. **(a)** During the encoding phase, information data is converted to a pattern that can be encoded on a MNP. Encoding phase compensates for the loss of individual bits of a microneedle patch over time and ensures error correction up to a certain percentage of bit corruption. Once the type of information data to be recorded is determined, it is translated to a binary string and then to information bits. Since the system is prone to unforeseeable errors such as missing bits from environmental trauma, temporal decay of fluorescent dye, and false positive signal from background noise, redundancy is added to the information bits using Reed-Muller error correcting code. Then, the generated string is mapped to a 2D pattern that fits in a template with four corners reserved for orientation. Encoding bits are arranged sequentially from top left to bottom right to generate an initial encoded pattern. An encryption mask is also added to ensure the privacy of personal medical data. **(b)** Decoding phase correctly translates acquired raw image back to the medical information that was originally recorded on patients during encoding phase. Decoding phase takes potential spatial imperfectness into consideration and makes a robust image recognition system based on deep learning (DL). Raw image is binarized using a DL-based image binarization network. Raw RGB image is converted to a BnW binary image, rectified to an axis-aligned and upright square geometry, and cropped and rotated to identify the MNP region by finding a minimum area rectangle. It is then fed into a DL-based image recognition network. The recognized binary array is re-oriented, and encryption step is reversed. Array is remapped into binary units for the Reed-Muller error correction decoding step and converted back to the corresponding string. Finally, it is translated back to the corresponding medical information text and is retrieved on a screen. **(c)** RM ECC adds redundancy to information bits so the transmitted message can be accurately recovered even when some bits are erroneously flipped. RM ECC corrects independent, non-block-based binary bits and is a good option for the OPMR system because spatial correlation between individual microneedle bits cannot be assumed for OPMR MNPs, and this would ensure a reliable long-term data retrieval. **(d)** Adding a known and fixed encryption pattern ensures the privacy of personal medical data of the OPMR system. **i**. The number of orientation bits are determined by subtracting encoding bits as per RM code from the total number of bits on an MNP. The orientation bits are allocated at four corners of the MNP with the bottom right corner OFF. **ii**. A pattern is first generated as a 2D array with roughly half ON-bits and half OFF-bits. **iii**. An encryption mask is added to the initially generated pattern. **iv**. After randomly flipping pixels on the raw encoded pattern, the encrypted pattern will consist of half ON and OFF pixels on average, which makes the recognition system robust to any patterns during the decoding step.

Our OPMR technology encodes information by imprinting two-dimensional (2D) patterns on MNPs, leveraging the binary feature of microneedle bits (that is, a microneedle (bit) is either present (ON) or absent (OFF)). The OPMR dye, once deposited in the skin, may experience NIR signal reduction due to phagocytic clearance, photobleaching or physical damage such as injury or scarring. To mitigate these potential data corruptions, our system features (1) an error correction scheme that introduces redundancy to compensate for temporal signal decay and (2) deep learning-based image processing to ensure reliable pattern readings despite spatial variations. The overall pipeline consists of two phases: an encoding phase and a decoding phase (Extended Data Fig. 4a,b).

**Fig. 3:**
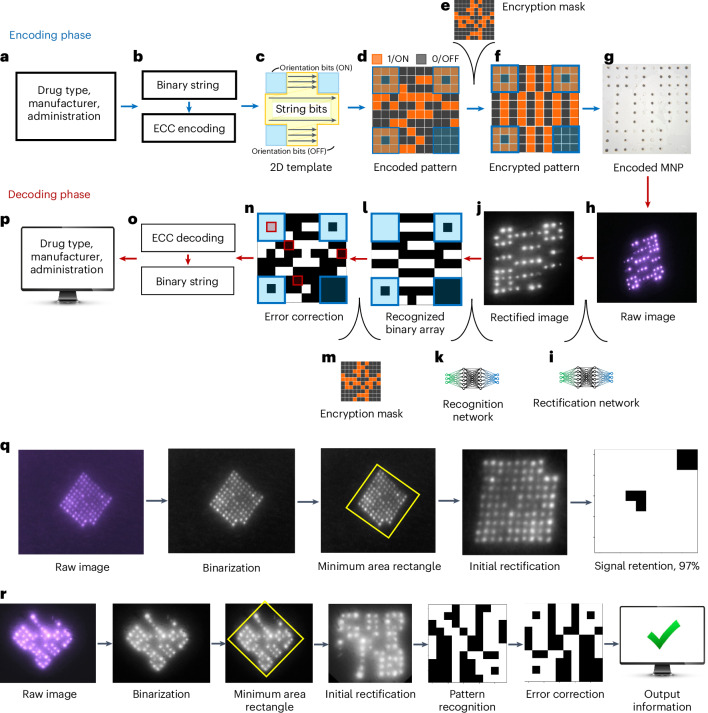
Deep learning-based networks allow parameter-free encoding and decoding of the OPMR. **a**, Examples of medical information that can be encoded on an OPMR MNP. **b**, Information data are converted to an encoded binary string before ECC. **c**, Binary information data are encoded following a 2D template. **d**, The 2D array becomes an encoded pattern. **e**, An encryption mask is applied for patient privacy. **f**, The encrypted pattern is generated for MNP encoding. **g**, Encoded MNP is fabricated. **h**, Decoding phase begins with raw image acquisition. **i**, Raw image is initially rectified via a deep learning-based rectification network. **j**, Rectified image is in a black-and-white square format. **k**, Bits are recognized by a deep learning-based recognition network. **l**, Recognition network outputs a binary array. **m**, Encryption step is reversed by removing the encryption mask. **n**, Error bits are identified. **o**, Error bits are corrected. **p**, Encoded binary string is translated back to the original information and output on a screen. **q**, Signal retention analysis quantifies the number of detected NIR bits for 96-bit MNPs. **r,** Pattern decodability analysis decodes patterned MNPs and evaluates whether they were decoded successfully or not.

The encoding phase was developed to mitigate potential MNP bit losses that may cause false representation of a pattern over time, and thereby provide temporal robustness. During encoding, information of interest gets converted to a pattern that can be encoded on a MNP. First, the information to be recorded on the patient was determined (Fig. 3a). Then, it was translated to an encoded binary string (Fig. 3b) with an error correcting code (ECC). The ECC added redundancy to the information bits as a means to defend against data corruption and therefore ensure reliable long-term information recovery^35^. Among the many types of ECCs, a Reed–Muller (RM) code^36,37^ (Extended Data Fig. 4c) was selected for the OPMR due to its suitability for correcting independent, non-grouped errors and its powerful encoding capacity of millions and billions of information combinations accompanied by its predetermined error correction capabilities (Table 1). For example, 128 different patterns can be generated with a 10 × 10 MNP and accurately decoded even when 15 error bits are present. Similarly, a 17 × 17 MNP can encode 137.4 billion different patterns and correct 31 error bits. This means billions of different patterns can be generated and used for encoding different medical information with a patch size of only 2 cm^2^.

**Table 1:** Information capacity of 2D MNP array based on RM ECC

Array size	Patch size	Total bit number	Orientation bits	Encoding bits	RM(*r*, *m*)	Information units	Number of pieces of encodable information	Correctable error bits
10 × 10	1 cm × 1 cm	100	36	64	RM(1, 6)	7	128 (that is, 2^7^)	15
12 × 12	1.2 cm × 1.2 cm	144	16	128	RM(1, 7)	8	256 (that is, 2^8^)	31
12 × 12	1.2 cm × 1.2 cm	144	16	128	RM(2, 7)	29	536.8 million (that is, 2^29^)	15
17 × 17	1.7 cm × 1.7 cm	289	33	256	RM(1, 8)	9	512 (that is, 2^9^)	63
17 × 17	1.7 cm × 1.7 cm	289	33	256	RM(2, 8)	37	137.4 billion (that is, 2^37^)	31

RM(*r*, *m*) denotes the RM code of order *r* and length 2^*m*^ (0 ≤ *r* ≤ *m*).

Once the encoded binary string was generated, it was mapped into a 2D pattern with a fixed orientation (Fig. 3c). The generated patterns consist of 1-bits (ON) where microneedles are filled with NIR dye and 0-bits (OFF) where microneedles do not contain any fluorescent dye (Fig. 3d). Next, an encryption mask was added to the encoded pattern to ensure the privacy of personal medical data (Fig. 3e and Extended Data Fig. 4d). The encrypted pattern (Fig. 3f) was then encoded on a physical MNP. The encoded MNP was fabricated by selectively loading dye to only the ON-bit needles, yielding roughly 50% ON-bit and 50% OFF-bit needles (Fig. 3g).

## Deep learning networks for spatially robust decoding

**Extended Data Fig. 5:**
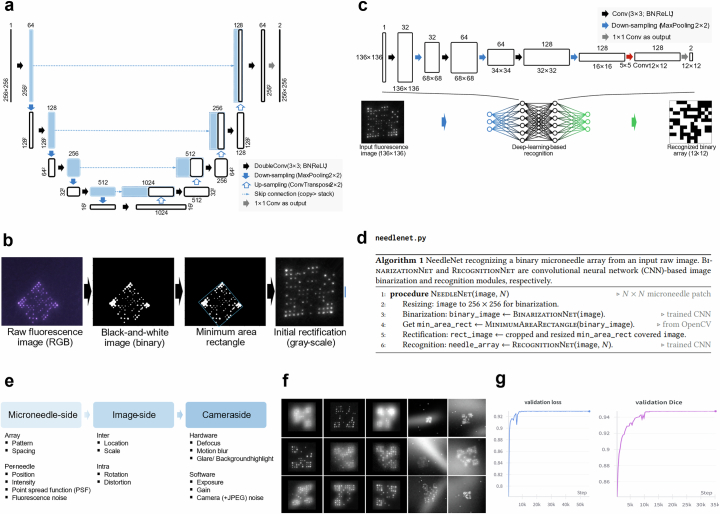
Deep learning networks for image binarization, rectification, and generation. **(a)** Image binarization network structure uses a U-Net adapted from an off-the-shelf Convolutional Neural Networks (CNN) for Biomedical Image Segmentation,. This is an image-based ConvNet, which is light and fairly accurate and easier to train with a certain amount of training examples. (DoubleConv: double convolutional layers; BN: batch normalization; ReLU: Rectified Linear Unit as the nonlinear activation function; and Skip connection: input or outputs from previous layers directly copied and then stacked as the input of current layer.) **(b)** 2D array region is rotated, cropped and resized a target size after the binarization step and before the rectification step. A rectangle with the minimum area that covers all the white bits of the 2D array region is generated. The final crop size is 35% larger than the size of minimum-area rectangle while preserving the center of the rectangle as a reference point. **(c)** A convolutional neural network (CNN)-based network structure is used for the image recognition model. (Conv: convolutional layer; BN: batch normalization; and ReLU: Rectified Linear Unit as the nonlinear activation function.) **(d)** RecognitionNet requires the microneedle patch size (NxN) as the input, while BinarizatioNet is independent of the microneedle patch size. **(e)** 650,000 simulated synthetic and paired train models were constructed for robust recognition network. Simulated fluorescence images were generated with potential image variations in three levels: 1) the quality of microneedles, 2) image acquisition, and 3) camera hardware and software. The previously used rectification network was applied to these synthetic models to output paired results, which were then input to a convolutional neural network (CNN)-based recognition network for it to learn the mapping of rectified images to binary arrays. **(f)** Examples of synthetic patch images with distortion, rotation, defocusing, motion blur, increased background noise, lowered contrast, and more. **(g)** The validation performances of the image binarization U-Net and image recognition CNN are 0.9297 and 0.9473, respectively, in terms of Sørensen–Dice coefficient (scale of 0 to 1; higher is better). The left plot shows the validation loss of the image binarization U-Net, and the right plot shows the validation loss of the image recognition CNN. The U-Net (for image binarization) and CNN (for image recognition) models resulted in signal retentions over 98% over 12 weeks with real-world pig images without fine tuning.

The spatial distribution of fluorescent dyes makes the OPMR system susceptible to bit distortions. The decoding phase was developed to compensate for these spatial variations between captured bit signals and to ensure spatial OPMR robustness. The decoding phase begins with the acquisition of raw images (Fig. 3h). Once acquired, each raw image was rectified to a square, binary format using a deep learning-based rectification network (Fig. 3i), which involves a U-Net-based^38^ binarization network (Extended Data Fig. 5a) that converts the raw red–green–blue (RGB) image to a black-and-white binary image and a minimum area rectangle function (Extended Data Fig. 5b) that finds, crops and rotates the MNP region. After rectification (Fig. 3j), the image was fed into a deep learning-based recognition network (Fig. 3k), developed by training a convolutional neural network^39^ (Extended Data Fig. 5c,d) with 650,000 synthetic images (Extended Data Fig. 5e–g). With these two deep learning steps, a raw image was successfully converted to a binary array (Fig. 3l). At this point, the binary array may have a corrupt pattern due to undetected or falsely detected signal bits. For accurate pattern decoding, the encryption mask was removed (Fig. 3m), and the deciphered pattern (Fig. 3n) was corrected with the RM ECC before being converted back to a binary string (Fig. 3o). The array was then, finally, translated and retrieved on a screen (Fig. 3p). The entire encoding-to-decoding workflow is completely automatic, requiring no user input or manual threshold manipulations due to the ‘end-to-end’ nature of this machine learning approach.

## Long-term information data preservation in swine

**Extended Data Fig. 6:**
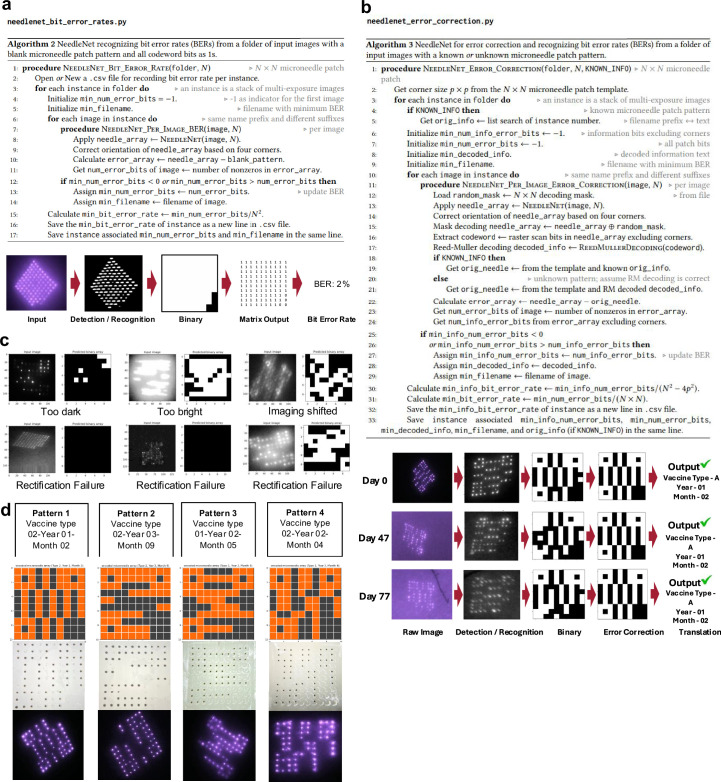
MNP signal retention, pattern decodability, and information recognition. **(a)** ‘needlenet_bit_error_rates’ code for automatic image processing system is programmed to analyze signal retention for 96-bit MNPs. It quantifyies the number of NIR bits that are preserved and detected for 96-bit MNPs and output the number as ‘signal retention %’. Raw fluorescence RGB image is converted to gray-scale and then to BnW, of which a minimum area rectangle tool rotates, crops, and resizes to a target size for an efficient recognition. Each bit is then recognized as either ON or OFF bits to find the percentage of ON-bits out of the 96 bits that were originally transferred on day 0. It is output as bit error rate, which is convertible to the signal retention %. **(b)** ‘needlenet_error_correction’ code is programmed to decode patterned MNPs and translate the NIR images of patterned MNPs back to the medical information that was originally encoded. Raw RGB image is converted to greyscale using DL-based binarization networks and cropped and rotated using the minimum area rectangle function. Each bit is recognized as either ON or OFF bit using DL-based recognition networks. Error bits are corrected by the RM ECC, and the corrected pattern is translated to information data. If this retrieved information accurately matches the original information data that was encoded on the MNP, then the pattern is processed as ‘successfully decoded’. **(c)** Initial challenging cases that were used as a guidance to improve simulations for training a robust image recognition network. Main causes of initial image recognition failure are rectification errors due to severe boundary noise, image distortion, and too large or too small global histogram threshold. Simulated spatial variations (e.g., under- and over- exposure, high background noise, high brightness variances) improved the image recognition network. **(d)** Four different 10×10 patterns were encoded on MNPs for the signal retention and information preservation evaluations in swine. Random medical information was assigned to each pattern.

Signal retention and pattern decodability were analysed longitudinally in a live pig model, as swine skin is known to be a close mimic of human skin^33^. For signal retention (Fig. 3q), the number of NIR bits that were preserved in the skin was quantified over time (Extended Data Fig. 6a). Each of the ON and OFF bits was detected to find the percentage of ON bits out of the total transferred bits, and this value was output as the signal retention percentage. For pattern decodability (Fig. 3r), patterned MNPs were decoded and outputs were compared to the intended encoded information (Extended Data Fig. 6b). If the retrieved information accurately matched the original information, then the pattern was designated as successfully decoded; otherwise, it was designated as unsuccessful (Extended Data Fig. 6c).

**Fig. 4:**
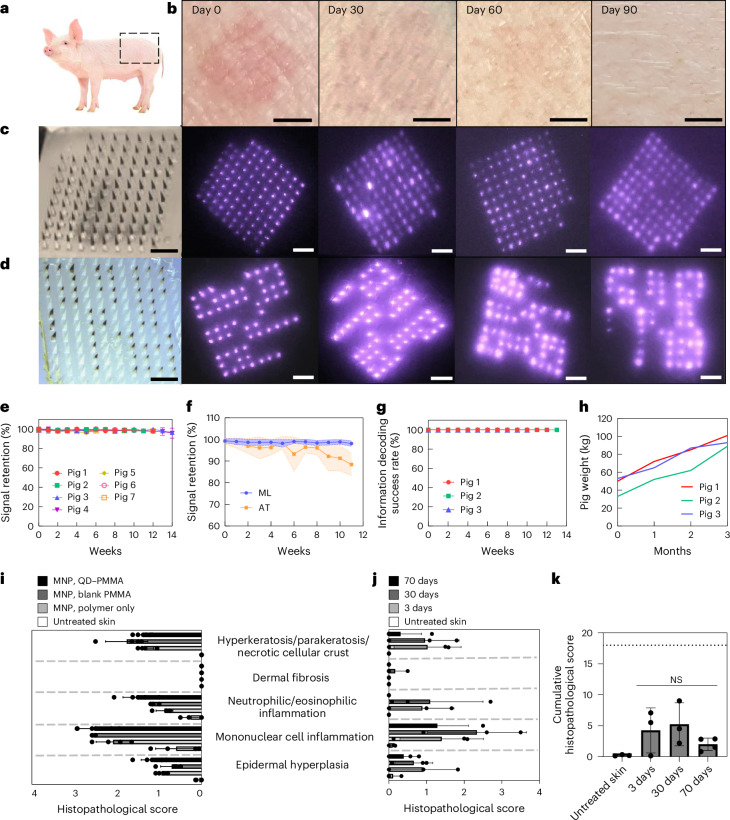
Long-term efficacy of OPMR in a swine model. **a**, MNPs were applied on the flank area of Yorkshire pigs. **b**, OPMR dyes deposited in the pig skin are invisible to the naked eye. Scale bars, 5 mm. **c**, NIR signals of 96-bit MNPs remain detectable for three months in pigs. Scale bars, 2 mm. **d**, NIR signals of patterned MNPs remain decodable for three months in pigs. Scale bars, 2 mm. **e**, NIR signal retention of 98.44% at 12 weeks in pigs; *n* = 24 MNPs across seven pigs. **f**, Machine learning (ML)-based custom image processing system outperforms adaptive threshold (AT) algorithm; *n* = 20, s.d. **g**, A 100% information decoding success rate occurred for 12 weeks in pigs; *n* = 21 MNPs across three pigs. **h**, Pig weights more than doubled during the three-month monitoring period**. i**, Histopathological scoring of pig skin with no treatment and with MNPs loaded with polymer only, PMMA microparticles and QD–PMMA microparticles; *n* = 4–6 MNPs per group, six slides per MNP. (Untreated skin and QD–PMMA MNPs had *n* = 5 biological replicates; the blank PMMA MNP and polymer-only MNP had *n* = 4 biological replicates; six tissue samples per biological replicate, s.d.) **j**, Histopathological scoring of untreated pig skin and pig skin with QD–PMMA MNP applied, at 3, 30 and 70 days after application; *n* = 3 MNPs per group, six slides per MNP. (Untreated skin, 3 days and 30 days had *n* = 3 biological replicates; 70 days had *n* = 4 biological replicates; six tissue samples per biological replicate, s.d.) **k**, Cumulative histopathological scoring shows a brief increase for the QD–PMMA MNP group that decreases over time (dotted line shows the maximum total score of 18). (Untreated skin, 3 days and 30 days had *n* = 3 biological replicates; 70 days had *n* = 4 biological replicates; six tissue samples per biological replicate. One-way analysis of variance (ANOVA); confidence interval, 95%).

MNPs were applied on the flank areas of Yorkshire pigs (Fig. 4a) and imaged weekly for three months. For signal retention, a total of 24 96-bit MNPs were applied on seven pigs. The dyes were invisible to the naked eye throughout the three-month monitoring period, resulting in completely indistinguishable patch application sites (Fig. 4b); however, the NIR signals remained visible during the entire monitoring period (Fig. 4c). For pattern decodability, a total of 21 patterned MNPs with four randomly selected 10 × 10 patterns (Extended Data Fig. 6d) were applied to three different pigs and imaged weekly for three months (Fig. 4d).

The signal intensity of these applied patches decreased over time^28^ (Fig. 2m), but with the scope of the camera and image acquisition (Extended Data Fig. 1a–c), NIR bits remained detectable, resulting in signal retentions of 98.69 ± 1.31% at 4 weeks, 98.35 ± 1.18% at 8 weeks and 98.44 ± 1.23% at 12 weeks (Fig. 4e). The number of error bits was well within the 15% correctable error bit threshold of the RM code of choice (Table 1). This completely automatic bit counting system processed 96-bit MNP images at an average speed of 0.043 second per image (*n* = 10,639 images). These machine learning-assisted results compared favourably to the adaptive threshold algorithm used previously in terms of both bit detection and precision (*n* = 20; Fig. 4f). For information preservation, all 21 patterned MNPs were decoded successfully across all three pigs over three months (Fig. 4g). This shows that our RM ECC successfully corrected the 1–2% of bit loss and retrieved accurate information over time. All 100% of the MNP footprints read out correct information despite evident spatial distortions caused by animal growth from 45.33 ± 10.78 kg to 94.33 ± 6.11 kg and epidermal cell turnovers during the three months (*n* = 3)^40,41^ (Fig. 4h). This completely automatic pattern decoding system analysed 10 × 10, patterned MNP images at an average speed of 0.066 second per image (*n* = 2,385 images) on a laptop with an Intel Core i7 tenth generation processor, suggesting that automated decoding is unlikely to be a noticeable source of delay in an OPMR workflow.

## Biocompatibility of the OPMR system

**Extended Data Fig. 7:**
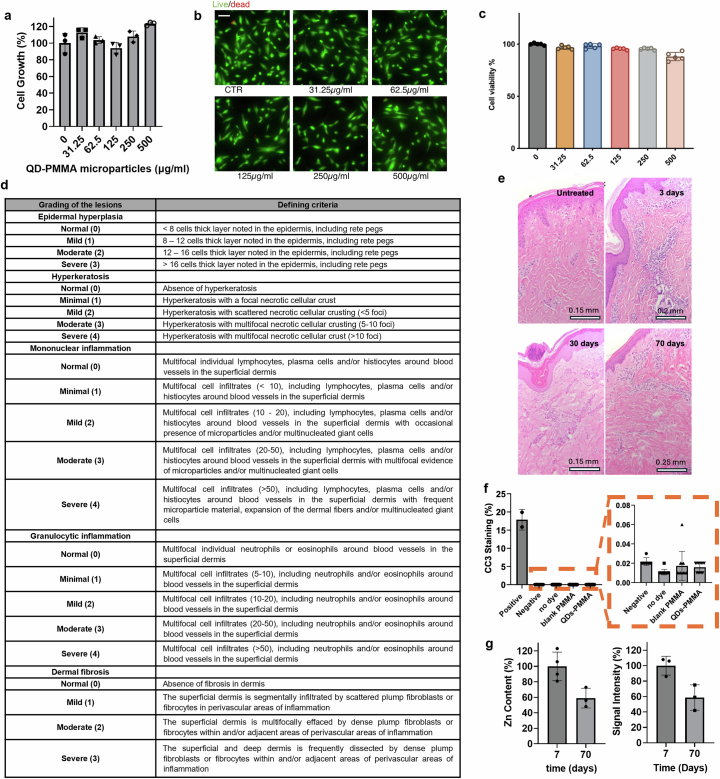
Safety evaluation of QD-PMMA loaded MNPs. **(a)** The cytotoxicity of the OPMR dye (QD-PMMA) was analyzed using HeLa cells. (n = 3, S.D., cytotoxicity test performed once.) When different concentrations of QD-PMMA microparticles were incubated for 20 hours, there was no effect on the cell growth % (**b)** The cytotoxicity of the QD-PMMA was also analyzed using adult human dermal fibroblasts that were incubated with QD-PMMA for 20 hours and assessed for toxicity via: Live/Dead assay, which uses green-fluorescent calcein-AM to indicate live cells and red-fluorescent ethidium homodimer-1 to stain dead cells, and the CCK-8 assay, which quantifies the metabolic activity of viable cells. (Scale bar=100 µm) **(c)** All QD-PMMA concentrations exhibited cell viability above 85%, indicating that the QD-PMMA microparticles are not cytotoxic, n = 5, S.D. **(d)** Grading criteria used for microscopic lesions seen in histopathological pig skins after MNP applications. **(e)** Skin sections were retrieved 3, 30, and 70 days post OPMR-MNP application and were processed and stained with hematoxylin and eosin for biocompatibility analyses for histopathological evaluation, showing mild to moderate subacute inflammation in the superficial dermis at day 3 post-application, and this dermatitis was minimal at day 30 and 70 post-application. At day 3 post-application, the dermis at the sites of microneedle injections was often infiltrated by histocytes, eosinophils, neutrophils, and occasional multinucleated foreign body giant cells, scattered around blood vessels. The epidermis at the sites of injections was slightly hyperplastic and/ or hyperkeratotic, containing nucleated keratin flakes, necrotic cellular debris (depicted at day 30 post-application). Groups: untreated skin, 3 days, 30 days, n = 3 biological replicates (samples from 3 different pigs), group 70 days, n = 4 biological replicates (samples from 4 different pigs), 6 tissue samples per biological replicate. **(f)** Quantification of CC3 staining of pig tissue with no treatment, with MNP applications loaded with just PVA/PVP polymer, blank PMMA microparticles, and QD-PMMA microparticles were applied 3 days prior to skin excision. To study if the OPMR system activates cell apoptotic mechanisms, tissue samples were stained with cleaved caspase-3 (CC3). And quantitative analysis showed no differences in the apoptotic cell % between the control and experimental groups, indicating there was no signal of immunoreactivity in the skin sections. n = 4 biological replicates (samples from 4 different pigs), 6 tissue samples per biological replicate, S.D. **(g)** To confirm the QD clearance from skin tissue, the zinc (Zn) content in pig skin 7 days and 70 days post OPMR-MNP application was evaluated using inductively coupled plasma optical emission spectrometer (ICP-OES, Agilent ICP-OES 5100 VDV) analysis after excising the tissue and dissolving it in Aqua Regia medium (nitric acid: hydrochloric acid 1:3). As a result, we observed 41 (±12.77) % of QD clearance at 70 days post-application as shown below. This reduction in the measured Zn content was conforming with the amount of signal reduction of the applied patch, which suggests the signal decrease over time is attributed to the QD clearance from the skin tissue. n = 3, biological, S.D.

To understand the long-term biocompatibility of the OPMR, the cytotoxicity of the OPMR dye was first examined in vitro with no indication of cytotoxicity (Extended Data Fig. 7a–c). Next, local inflammatory and cellular responses in the area of MNP administration were examined. Upon MNP application, a mild erythema was initially observed and disappeared within 30 min (Fig. 2f). For histopathological evaluations, tissue sections applied with nothing and with MNPs containing polymer, blank PMMA microparticles and QD–PMMA microparticles were excised 3 days post-application. Cutaneous lesion scores for the QD–PMMA MNP group were comparable in severity to the other MNP control groups (Extended Data Fig. 7d), suggesting that the observed lesions were induced by trauma from needle penetration itself, irrespective of the PMMA or QD dye content^42^ (Fig. 4i). Skin sections retrieved 3, 30 and 70 days after QD–PMMA MNP application (Extended Data Fig. 7e,f) exhibited minimal to mild dermal inflammation at both days 30 and 70, a complete absence of hyperkeratosis at day 70 and no evidence of fibrosis at any examined time point (Fig. 4j). There were no clinically or statistically significant differences between cumulative histopathological scores of untreated and MNP-applied skin samples (Fig. 4k). Lastly, successful QD clearance from the skin tissue over time was also observed (Extended Data Fig. 7g).

## Co-delivery of OPMR and mRNA vaccine for SARS-CoV-2

**Fig. 5:**
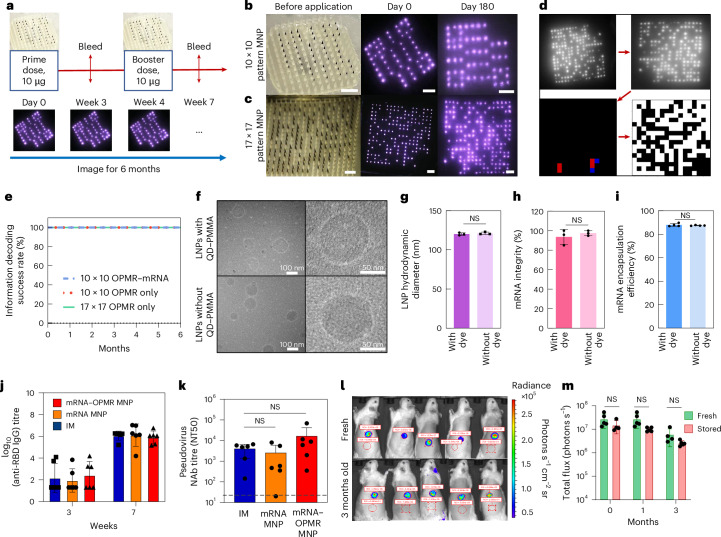
MNPs that co-deliver the OPMR and potent mRNA vaccine successfully record information and induce immunogenicity in rats. **a**, Prime and booster doses were applied on days 0 and 28, respectively, with OPMR–mRNA MNPs to assess the co-delivery of OPMR and mRNA. **b**, Optical image of a 10 × 10 patterned OPMR MNP and its NIR footprint in rats over 180 days. Scale bars, 2 mm. **c**, Optical image of a 17 × 17 patterned OPMR MNP and its NIR footprint in rats over 180 days. Scale bars, 2 mm. **d**, All patterns exhibited a correctable number of error bits and were successfully decoded. **e**, All three groups (10 × 10 patterned OPMR MNP, 10 × 10 patterned OPMR–mRNA MNP and 17 × 17 patterned OPMR MNP) were successfully decoded over six months; *n* = 5–6. **f**, Cryo-TEM images of vaccine solution show intact, monodispersed mRNA-LNPs with and without OPMR dye. (TEM performed once.) **g**, DLS analysis shows comparable LNP sizes with and without OPMR dye; *n* = 3. **h**, Fragment analyser analysis shows comparable mRNA integrities with and without OPMR dye; *n* = 3. **i**, Ribogreen assay shows comparable mRNA encapsulation efficiencies with and without OPMR dye; *n* = 5. **j**, IM control group, mRNA MNP group and mRNA–OPMR MNP group induce comparable IgG titre levels in rats; *n* = 6. **k**, IM control group, mRNA MNP group and mRNA–OPMR MNP group induce comparable post-boost pseudovirus neutralizing antibody (NAb) titre levels in rats. Naive rat response is shown as a dashed line; *n* = 6. **l**, OPMR–mRNA MNPs encoding luciferase were stored at room temperature for three months and applied to rats for a shelf-life study, and their luciferase expressions were quantified using an in vivo imaging system. Red circles are selected regions of interest (ROI) to measure the radiance. **m**, Luciferase expressions of MNPs stored for one month and three months are comparable with those of fresh patches; *n* = 5. NT50, levels of 50% neutralizing titer.

**Extended Data Fig. 8:**
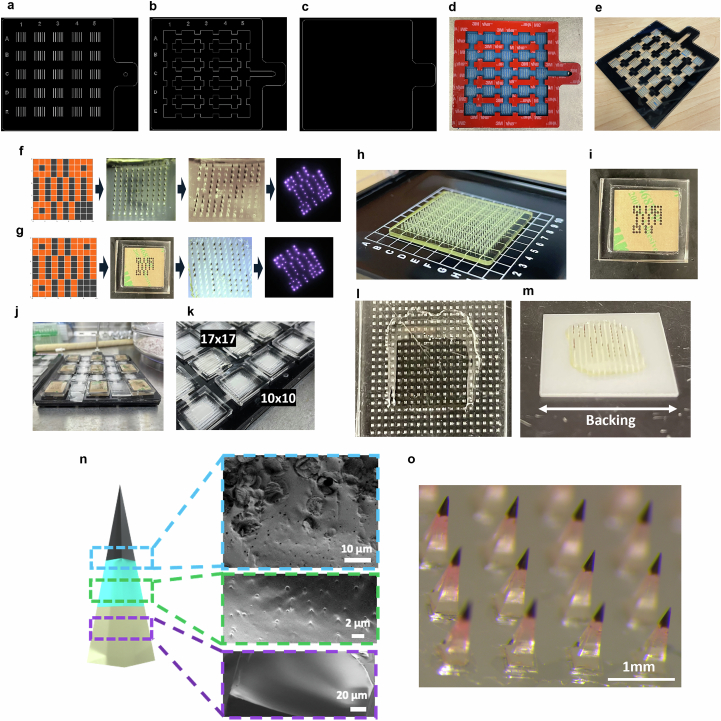
Fabrication of MNPs with OPMR and mRNA-LNPs. Vacuum through devices were custom designed and fabricated; **(a)** Top, **(b)** middle and **(c)** bottom layers of the device were designed using 2D CAD modeling. The top and bottom pieces were made by laser cutting 1/8’-thick Acrylic sheets (McMaster-Carr 8589K41), and the middle piece was made by laser cutting 0.045’-thick Acrylic foam adhesive sheet (McMaster-Carr 1630N24). The middle layer was adhered to the top layer first **(d)**, and then the bottom layer was adhered to complete the assembly **(e)**. Patterned MNPs were fabricated either by manually knocking out QD-loaded needles **(f)** or by selectively loading QDs using a mask **(g)**. These two methods did not make a difference in signal transfer or signal longevity results. Patterned masks can be created by hole-punching or laser-burning during MNP fabrications. Other viable approaches to fabricate patterned MNPs could involve molds that consist of adjustable pins. Once a pattern is generated, a robot could push selected pins to create a positive mold representing the desired pattern in real time. The adjustable pin arrays could also directly press against pre-made full array MNPs to punch out specific needles. **(h)** Master molds of 20x20 needle arrays are 3D printed for negative PDMS mold fabrication. **(i)** A patterned mask is placed on top of the PDMS mold. **(j)** QD-PMMA solution is dispensed on the masked PDMS under vacuum to selectively load the dye into the needle tips in a pattern. **(k)** The mRNA-LNP-polymer solution is then added to the mold for the mRNA-OPMR MNP fabrication. **(l)** The footprint of dispensed mRNA-LNP-polymer solution while drying. **(m)** Once the polymer dries, a Delrin backing is attached to the back of the MNP, and the patch is removed from the PDMS mold to be further dried in a desiccator under vacuum for 48 hours. Once an MNP is applied to the skin, the microneedles are designed to readily dissolve within minutes, simultaneously delivering a clinically relevant dose of mRNA vaccine and an NIR pattern that represents corresponding medical information (e.g., vaccine type, manufacturer, vaccination date) to the dermis layer, producing effective immunogenicity and long-term information recording on patients. **(n)** Cross-sectional scanning electron microscopy (SEM) images of an OPMR-mRNA-MNP needle near the tip showing the OPMR dye (QD-PMMA microparticles; 10 µm), near the mid-body region where mRNA vaccine solution is loaded and at the backing of the needle where the PVA-PVP material is showing a smooth surface. Imaging was performed once. **(o)** Co-loaded OPMR-mRNA MNP needles will have OPMR dye concentrated at the needle tips and vaccine loaded in the needle bodies as depicted with pink color dye.

The OPMR MNP can co-deliver therapeutics to patients by adding a secondary cargo^43–46^ (Extended Data Fig. 8). We demonstrated efficient and safe co-delivery of the OPMR and an mRNA vaccine encoding SARS-CoV-2 receptor-binding-domain (RBD) spike protein, encapsulated in LNPs in a rat model (Fig. 5a).

**Extended Data Fig. 9:**
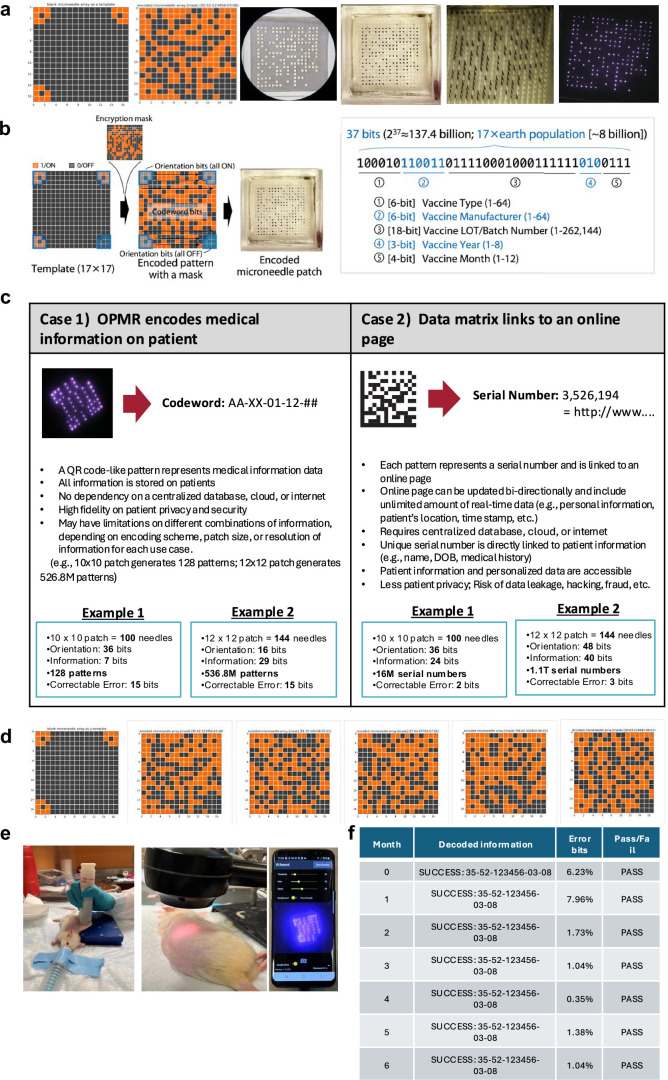
Evaluation of information encoding and decoding with OPMR MNPs. **(a)** A 17×17 pattern demonstrates the feasibility of recording billions of different patterns. Eight bits in each corner were assigned for patch orientation. An encrypted pattern was generated for encoding. A mask was fabricated using laser cutting. QD fluorescent dyes were selectively loaded to the PDMS negative mold. Patterned MNP was demolded from mold. The patterned MNP was applied to rats for long-term OPMR evaluations. **(b)** Encoded information on a microneedle patch can be simple (e.g., vaccine type, manufacturer, LOT/batch number, vaccination year and month), or complex (e.g., drug package inserts, active ingredient, warnings and precautions, prescribing information). Our current strategy of storage uses information bits as indexing numbers to encode 1) medical information by separating 37 bits into blocks, of which each is an index for a piece of information, or 2) unique identifier for each patient, which can cover the entire world’s human population and tens of generations after that with a 1.7 cm x 1.7 cm MNP. We can also use these indexing numbers to look up a library of hundreds of pages of documents. The information to encode can be determined as per use case while maximizing the usage of the on-patient bits. **(c)** Encodability can potentially be enhanced by utilizing each pattern as a ‘data matrix’, which assigns a serial number per pattern that can be linked to an online page. With the ‘data matrix’ approach, 1.1 trillion different serial numbers can be encoded with a 12 x 12 patch. **(d)** Five randomly selected representative 17×17 patterns are shown as examples. **(e)** Application and imaging of MNP on a rat using our custom applicator and imaging system. **(f)** 17×17 OPMR MNP encoding for ‘35-52-123456-03-08’ was decoded correctly for a 6-month monitoring period. The number of error bits fluctuate over the course of monitoring period due to rat skin irritation, rat fur interfering with the signal, and other environmental factors, but these error bits are successfully corrected with RM ECC.

First, to evaluate the performance of the OPMR when co-delivered with mRNA encapsulated in LNPs, the pattern decodability of the OPMR was studied with and without mRNA-LNPs. For this test, 10 × 10 patterned MNPs (Extended Data Fig. 6d) with and without mRNA-LNPs (Extended Data Fig. 9a–d) were applied to Wistar rats and imaged for six months (Extended Data Fig. 9e). A 17 × 17 patterned MNP group was added to demonstrate the feasibility of recording billions of different patterns in the long term (Table 1). The footprints of both 10 × 10 (Fig. 5b) and 17 × 17 (Fig. 5c) MNP groups remained detectable and successfully decodable (Fig. 5d) during the entire six-month monitoring period (*n* = 5–6; Fig. 5e and Extended Data Fig. 9f). These 100% success rates indicate that a long-lasting OPMR is feasible with mRNA-LNP delivery.

**Extended Data Fig. 10:**
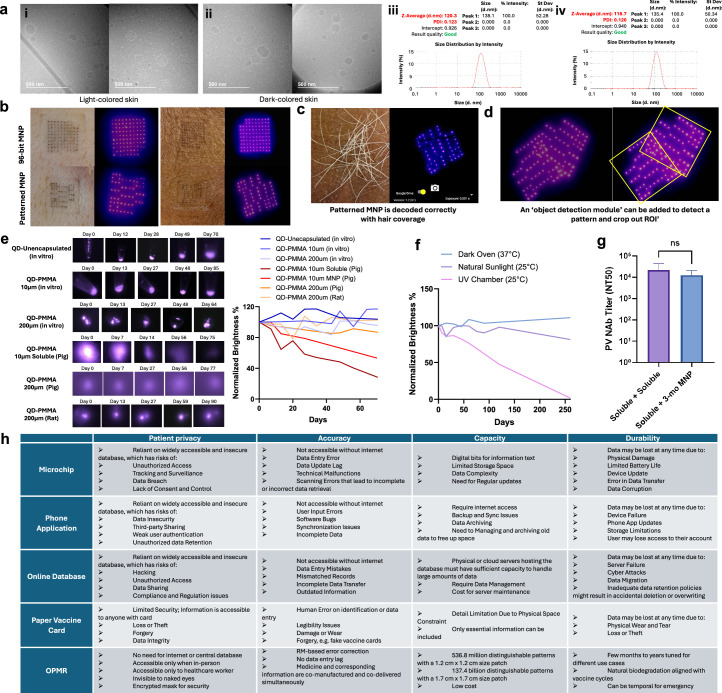
Co-delivery of OPMR and mRNA-LNP vaccine encoding for SARS-CoV-2. **(a)** Cryo-TEM images of vaccine solution show intact, well-dispersed mRNA-LNPs with **(i)** and without **(ii)** the OPMR dye (TEM performed once). DLS analysis show comparable Z-averages of mRNA-LNPs with **(iii)** and without **(iv)** the OPMR dye (DLS performed for 3 replicates). **(b)** 96-bit MNPs and patterned MNPs were applied to light-colored porcine skin and dark-colored human cadaveric skin to assess potential light absorbance, they exhibited comparable BER of 0-1 %, and successful pattern decodability. This demonstrates that the pigment of skin does not substantially affect OPMR performance. **(c)** OPMR performance with obstructed viewing of the pattern due to hair coverage was tested to assess potential suboptimal results. NIR signals penetrated through, resulting in successful pattern imaging and decoding. **(d)** Regarding potential complications arising from patterns that are overlapped or in close proximity, a ‘bounding box’ feature can help the object detection module. Object detection module easily detects pattern without an additional bounding box, but reserving the outer-most edges of patterns will facilitate ‘minimum square area’ cropping for pattern isolation and detection. **(e)** Quenching of QD-PMMA and shelf-life of mRNA-OPMR MNP were quantified in different settings. Signal brightness of 1) unencapsulated QD particles, 2) 10 µm size QD-PMMA particles, and 3) 200 µm size QD-PMMA particles in PBS in Eppendorf tubes, 4) 10 µm size QD-PMMA particles and 5) 200 µm size QD-PMMA particles in a live pig’s dermis layer, and 6) 200 µm size QD-PMMA particles in a live rat’s dermis layer were assessed for 70 days. As a result, all the *in vitro* groups and *in vivo* rat group exhibited no reduction in QD signal brightness, whereas the *in vivo* pig groups including the MNP applied groups exhibited a gradual signal reduction over time. This signifies that the signal reduction over time is caused by the QD clearance from the skin tissue rather than quenching. **(f)** Signal brightness of 10 µm size OPMR dye in PBS in Eppendorf tubes in a dark incubator was quantified, when exposed to natural sunlight, and when directly exposed to UV light-only (UVP CL-1000 Ultraviolet Crosslinker, 365 nm) for 250 days. The signal brightness of QD particles remained unchanged for the dark oven group, was reduced by 18.85 % for the natural sunlight group, and was reduced by 98.27 % for the UV chamber group. This signifies that quenching is most affected by the UV exposure. **(g)** SARS-CoV-2 RBD mRNA-OPMR MNPs were applied after being stored in a desiccator at RT for 3 months. Rats were primed with an IM injection of 10 μg of fresh mRNA-LNPs soluble and boosted with the 3-month stored MNPs (n = 5, S.D.), and there was no notable difference compared to fresh soluble primed and fresh soluble boosted group (n = 6, S.D.). These results showcase a promising shelf-life of mRNA-LNP-OPMR MNPs. **(h)** The advantages of the OPMR technology is summarized in a table. Comparisons between microchips, phones, online databases, paper vaccine cards, and OPMR are detailed.

Second, to study the efficacy of mRNA vaccine delivery with the OPMR, the integrity of LNPs was first characterized with and without the OPMR dye in vitro. When analysed with cryo-transmission electron microscopy (cryo-TEM; Fig. 5f) and dynamic light scattering (DLS; Fig. 5g and Extended Data Fig. 10a), the mRNA-LNPs remained stable and monodispersed with average sizes of 121.20 ± 1.56 nm and 120.20 ± 1.67 nm with and without OPMR dye, respectively. Both mRNA strands and LNPs remained intact with mRNA integrities of 93.60 ± 7.72% and 97.40 ± 2.59% (Fig. 5h), and mRNA encapsulation efficiencies of 87.50 ± 0.32% and 87.95 ± 1.28% (Fig. 5i), with and without OPMR dye, respectively, as well. Next, to evaluate the mRNA vaccine delivery with the OPMR in vivo, three groups were tested for immunogenic responses: (1) a control group with intramuscular (IM) injections, (2) an mRNA MNP group loaded with vaccine only and (3) an mRNA–OPMR MNP group co-loaded with vaccine and OPMR. For these groups, prime doses were applied on day 0, and booster doses followed 28 days after (Fig. 5a). All three groups exhibited comparable post-boost immunoglobulin G (IgG) titre levels (*n* = 6; Fig. 5j) as well as pseudovirus neutralizing antibody titre levels (*n* = 6; Fig. 5k), indicating that the OPMR MNPs successfully provided non-inferior protection against SARS-CoV-2 compared to the IM controls. This demonstrates that co-delivery of effective mRNA therapeutics is feasible with our OPMR MNP technology.

Finally, to evaluate the shelf life of the OPMR–mRNA MNPs, MNPs loaded with mRNA encoding firefly luciferase (FLuc) and OPMR dye were stored at room temperature for three months and applied to rats at different time points. When the bioluminescence of the FLuc expression was quantified with an in vivo imaging system at 6 h post-application (Fig. 5l), no substantial differences existed between fresh patches and those stored for one month or three months (*n* = 5; Fig. 5m), highlighting the possibility of storing, distributing and applying these patches on-demand for mRNA therapeutics delivery and recording.

## Outlook

Here we developed a robust microneedle-based OPMR technology that can store information intradermally with excellent temporal and spatial robustness and an encoding capacity up to the billions. The demonstration of the co-delivery of the OPMR with reliable information retrieval and a potent mRNA vaccine exhibited in this work suggests a potential translation of the technology to clinical uses. In cases of emergency like in a pandemic or natural disaster, or at refugee or military camps, OPMR patches can be administered on-demand and can help healthcare workers make appropriate decisions on the follow-up dose administrations without patient confidentiality risks. To further strengthen the OPMR’s long-term reliability, different case scenarios (for example, pattern signal attenuation due to skin pigment, hair or pattern overlap; Extended Data Fig. 10b–d) for a prolonged period of time (for example, OPMR MNP stability for one year; Extended Data Fig. 10e–g) could be investigated in the future. Overall, this technology is readily applicable for any mRNA therapeutics, considering its compatibility with mRNA-LNPs and its large encoding capacity to complement the increasing number of mRNA therapeutics currently under development. As mRNA therapeutics aim to combat a wide range of preventable and incurable diseases, this OPMR technology presents an opportunity to bring healthcare equity one step closer to reality (Extended Data Fig. 10h).

## Methods

### QD encapsulation in PMMA microparticles

Commercial CuInS_2_/ZnS QDs were purchased from Strem Chemicals. PMMA (molecular weight, ~120,000) was purchased from Sigma-Aldrich. QD encapsulation was performed following the solvent emulsification evaporation technique. Some 100 mg of QDs and 100 mg of PMMA were dissolved in 2 ml dichloromethane (DCM). The QD–PMMA solution was then added to 20 ml of cold 1% w/v polyvinyl alcohol (PVA) solution and emulsified at 10,000 rpm for 1 min (T 18 digital ULTRA-TURRAX homogenizer, IKA). The resulting emulsion was immediately poured into 30 ml of 1% w/v PVA solution and stirred at 250 rpm overnight to allow the evaporation of the DCM. Following this, the solution was poured into a 50 ml centrifuge tube and centrifuged at 2,400*g* to collect the microparticles. The supernatant was discarded and the microparticles were washed three times by adding sterile deionized water followed by centrifugation. Finally, the particles were resuspended in a small amount of water and filtered through a 75 μm filter to remove large aggregates. The QD-microparticles were then dried and kept in the dark, under vacuum until use.

### Photoluminescence spectroscopy

Photoluminescence emission spectra were measured using a thermoelectrically cooled silicon camera (PIXIS100, Teledyne Princeton Instruments). Samples were prepared in quartz cuvettes by suspending QDs or QD–PMMA microparticles in cyclohexane. The samples were excited using a 532 nm laser (CPS532, Thorlabs). The emission was collected and focused using two silver-coated off-axis parabolic mirrors, and it was filtered through an 800 nm long-pass dielectric filter into a monochromator before imaging on the silicon camera. Photoluminescence quantum yield data were measured using a silicon photodiode (818-UV, Newport) coupled to a lock-in amplifier (SR830, Stanford Research), using chopped 405 nm laser excitation (LDM405, Thorlabs) and an optical integration sphere (RTC-060-SF, Labsphere)^47^.

### NIR fluorescence imaging

A universal serial bus (USB)-connected NIR fluorescence imaging system involving a custom light-emitting diode (LED) module that emits shorter-wavelength NIR light at 780 nm and a USB-connected camera module that captures the excited QD fluorescence image at longer-wavelength NIR light at >850 nm were used to image the QDs, with a photoluminescence intensity peak at 890–897 nm. An Android smartphone application ‘IR Record’ was custom developed to capture the OPMR NIR dye signal and can save images. The software is designed to take 30 consecutive images with six different exposure settings and five different gain settings. This bracket scanning method allows the capture of NIR signals with varying intensities over time. Among the 30 images, one image with the best reading results gets automatically chosen and processed. The total amount of required time to scan one OPMR patch on a patient is currently slightly over 2 min. However, further improvements can be made by pushing the scanning and recognition process to real time. To further advance the temporal aspect of the image-acquisition-to-image-recognition pipeline, (1) an ‘OPMR detection module’ that pinpoints the location of a relatively small patch, (2) an ‘auto-exposure module’ that acquires and stores one sufficient image for recognition instead of capturing a series of images with a wide window of exposure levels and (3) a ‘mobile-deployed decoding phase’ that processes everything on the fly can be added to our automatic system. With these implements, comprehensive scanning and recognition of numerous patterns will be executable in a more timely manner in real-world scenarios. In reality, we observed that the imaging environment (for example, ceiling lights in the surgical room of the pig facility, the height of the bed that the pigs were placed on and the distance between the bed and lights), the imaging personnel (for example, manual adjustment of the camera settings and distance away from the surface of the pig skin) and the pigs’ condition (for example, a scratch or fur on the pig skin) affect the quality of the images more than the actual quality of the NIR signals. These factors reflect real-world imaging scenarios more accurately as imaging will be performed by different healthcare professionals across different points of care.

### Polydimethylsiloxane mould fabrication

Positive master moulds were designed using a computer-aided design (CAD) software (SolidWorks, Dassault Systèmes) and custom manufactured on a five-axis computer numerical control (CNC) milling machine using tool steel blanks. Master moulds with smaller needle spacings that were not suitable for CNC machining were three-dimensionally (3D) printed with HTL resin and ultra-high-resolution 3D printers (Boston Micro Fabrication). These master moulds were used to generate negative moulds made of polydimethylsiloxane (PDMS; Sylgard 184, Dow Corning). The PDMS base and crosslinking agent were mixed according to the manufacturer’s instructions, poured onto the positive master mould and cured overnight at 60 °C. To create additional MNP positives, UV-curable Norland Optical Adhesive 61 (Cranbury) was filled into the PDMS negative moulds using a centrifuge at 3,234*g* for 1 min, placed in a UV-curing oven at room temperature for 20 min and manually removed.

### The mRNA-LNP solution synthesis

LNPs were synthesized to encapsulate mRNA^48–50^. Pseudouridine-modified mRNA encoding FLuc (TriLink BioTechnologies) and pseudouridine-modified mRNA encoding the SARS-CoV-2 spike protein (National Institutes of Health code/strain) with furin cleavage site deletion, two proline mutations and a trimerization foldon for stability (ACROBiosystems) were purchased. For the LNP synthesis, Lipid 5 ionizable lipid (heptadecan-9-yl 8-((2-hydroxyethyl)(8-(nonyloxy)-8-oxooctyl)amino)octanoate; Organix), 1,2-dioleoyl-*sn*-glycero-3-phosphoethanolamine (DOPE; Avanti), cholesterol (Sigma-Aldrich) and 1,2-dimyristoyl-*sn*-glycero-3-phosphoethanolamine-*N*-[methoxy-(polyethyleneglycol)-2000] (ammonium salt; C14-PEG2000; Avanti) were dissolved in ethanol at a molar ratio of 38.4:12.3:47.4:1.9, respectively. To prepare the LNPs, the ethanoic solution was rapidly added to and mixed with an mRNA solution buffered with citrate at pH 3 at volume ratio 3:1 (aqueous/ethanol). The ionizable lipid to mRNA weight ratio was set to 5, and the final mRNA concentration was 0.135 mg ml^–1^. All nucleic acids were stored at –80 °C and were allowed to thaw on ice prior to use. The LNPs were then dialysed for at least 2 h in phosphate buffered saline (PBS) at 4 °C in a 20,000 molecular weight cut-off cassette. For mRNA-LNP solution synthesis for MNP fabrications, LNPs were further dialysed in deionized water for an additional 2 h at 4 °C, and then concentrated to 320 µg ml^–1^ on an Amicon filter by centrifuging at 3,000*g*. Finally, the mixture was diluted to a polymer solution made of PVA (Mowiol 4-88; molecular weight, 31,000; Sigma-Aldrich) and polyvinylpyrrolidone (PVP; molecular weight, 10,000; TCI) to form the vaccine solution.

### OPMR microneedle fabrication

OPMR MNPs were fabricated using a centrifugation technique. To load the OPMR dye to the MNPs, 200 μl of an aqueous dispersion of QD–PMMA microparticles (3–10 mg ml^–1^) was dispensed on top of a 10 × 10 negative PDMS mould and centrifuged at 2,400*g* for 3 min to concentrate the microparticles at the needle tips. To ensure an even loading of the dye across the MNP, the mould was rotated 180° before adding another 200 μl of the dye dispersion and was centrifuged for another 3 min. One more round of 200 μl of the dye dispersion loading and centrifugation was done without rotating the mould this time. For the microneedle body and backing, 150 μl of 30% w/w PVA/PVP (1:1) solution was added and centrifuged at 2,400*g* for 5 min. An additional 500 μl of the PVA/PVP solution was added and centrifuged in two steps with a 4 h time interval in between to ensure a flat surface of the backing after drying. After drying at room temperature for 4.5 days, a Delrin acetal plastic backing with adhesive tape (0.508 mm thickness; McMaster-Carr) was attached to the back of the MNP before removal from the mould. Once removed, it was further dried under vacuum (–60 MPa) for 48 h. As a result, each MNP ended up with a maximum of 0.8 mg QD–PMMA.

FLuc mRNA–OPMR MNPs were fabricated via a two-step loading using a vacuum-through technique. PDMS moulds were placed on a vacuum-through device to load cargos into the mould using negative pressure, leveraging the air permeable characteristic of PDMS. As the first step, 1 ml of an aqueous dispersion of QD–PMMA microparticles (1.5 mg ml^–1^) with 0.5% w/w PVA/PVP was dispensed on the PDMS mould, and a vacuum (–85 MPa) was applied to the vacuum-through device overnight. As the second step, 200 μl of FLuc mRNA-LNPs mixed with PVA/PVP solution (1:320 mRNA to polymer ratio by weight^43^) was dispensed and left to dry overnight under vacuum. As the final step, 80 μl of 20% w/w PVA/PVP solution was dispensed to form the MNP backing and was left to dry overnight under vacuum. Once dried, a Delrin backing was attached for patch removal, and the removed patches were further dried in a desiccator under house vacuum for 48 h and until use. The mRNA-only MNPs were fabricated the same way excluding the first step. For the shelf-life studies, FLuc mRNA–OPMR MNPs were fabricated and stored in a desiccator at room temperature for months. For each test time point, a fresh batch of mRNA–OPMR patches was fabricated as a positive control.

Patterned SARS-CoV-2 mRNA–OPMR MNPs were fabricated via the two-step loading using the vacuum-through technique. To make patterned MNPs, laser-cut mask tapes (468MP PEI Adhesive Transfer Tape Sheet, 3M) with patterns were placed on 20 × 20 PDMS moulds. Then, the PDMS moulds were placed on a vacuum-through device to load cargos into each mould using negative pressure, leveraging the air permeable characteristic of PDMS. As the first step, 1 ml of an aqueous dispersion of QD–PMMA microparticles (1.5 mg ml^–1^) with 0.5% w/w PVA/PVP was dispensed onto the top of the mask on the PDMS mould, and a vacuum (–85 MPa) was applied overnight. Then, the mask was removed, and the mould surface was cleaned with an ethanol wipe. As the second step, 180 μl of vaccine solution made of SARS-CoV-2 RBD mRNA-LNPs (589 μg ml^–1^ encapsulated mRNA) mixed with PVA/PVP solution at a 1:320 mRNA-to-polymer ratio by weight was dispensed and spread on top of the mould, covering approximately 100 needles in the centre, and was left under vacuum overnight. As the final step, a Delrin backing was attached for patch removal, and the removed patches were further dried in a desiccator under house vacuum for 48 h and until use. As a control group, SARS-CoV-2 RBD mRNA MNPs without the addition of OPMR dye were fabricated; this group was fabricated the same way excluding the first step. For 17 × 17 patterned patches, no mRNA was loaded. Instead, after the dye loading, 1 ml of 20% w/w PVA/PVP polymer solution was added and spread across the 17 × 17 array to form the needle body and backing. The process to deliver a sufficient dose of mRNA vaccine with the MNP is shown in a design relationship plot in our recent publication^43^, created to meet dosing requirements in humans for common mRNA vaccines; it illustrates the relationship among single microneedle volume, microneedles per MNP and dose delivered in a single MNP. Using the needle volume and MNP loading efficiency from a study on anti-COVID RBD titre peaks, this model predicts the combination of volume and number of microneedles necessary to deliver the full Moderna (mRNA-1273) or Pfizer–BioNTech (BNT162b1) COVID-19 vaccine doses. The model predicts that 360 and 108 of the studied microneedles and formulations would deliver the full dose of the Moderna and Pfizer–BioNTech vaccines, respectively. MNPs containing a sufficient dose are less than 2 cm across.

### Scanning electron microscopy analysis

SEM was used to image the QD–PMMA microparticles and the OPMR and OPMR–mRNA MNPs. Samples were initially coated with a thin layer of Au using a sputter coater (Desk V, Denton Vacuum) and then imaged using high-resolution SEM (Zeiss Crossbeam 540 Scanning Electron Microscope and Focused Ion Beam, Zeiss).

### Ex vivo and in vivo MNP applications

All animal procedures were approved and performed under the guidelines of the Massachusetts Institute of Technology Committee on Animal Care. Three-month-old female Yorkshire pigs were provided by Cummings School of Veterinary Medicine. Six-week-old to eight-week-old female Wistar rats were purchased from Charles River. For the ex vivo MNP applications, hand application, a commercial spring applicator (Micropoint Biotechnologies) and custom-made applicators with a velocity upon impact ranging from 1,066–1,583 cm s^–1^ and holding force within the range of 0.16–1.98 MPa were tested on excised flank and hip skins of pig cadavers. For ex vivo human skin tests, donated cadaveric skin tissue samples (National Disease Research Interchange, Philadelphia, PA) were used. For in vivo MNP applications, a custom-made applicator with a velocity on impact of 1,407 cm s^–1^ and holding force of 1.1 MPa was used to apply patches on the flank and hip areas of Yorkshire pigs and on the back area of Wistar rats. Patches were applied for 5–10 min for pigs and 10–20 min for rats.

### Dye deposition depth assessment

To assess the dye deposition depth in the skin, skin tissues were excised after MNP applications, fixed in 10% formalin buffer for 48 h, transferred to 70% ethanol and then embedded in paraffin wax. Samples were sectioned at 5 μm width every 20 μm to retrieve 30 slices for one entire microneedle array for cross-sectional evaluations of the maximum needle penetration and dye deposition depths. The samples were stained with hematoxylin and eosin and analysed with Aperio software (Leica Biosystems). Furthermore, parts of the skin tissue were frozen and fixed in Optimal Cutting Temperature compound for cross-sectional imaging to detect the presence of the NIR bits in the dermis.

### Needle dissolution, bit transfer, signal retention and signal intensity analyses

To quantify microneedle dissolution as a function of different MNP architecture variables, MNPs were imaged before and after application using a Leica DFC450 optical microscope. The patches were placed in a transverse manner for imaging using LAS v.4.7 software. Microneedle length was calculated using ImageJ (National Institutes of Health) for >10 microneedles per MNP for 3–5 MNPs per group. For bit transfer and signal retention analyses, ImageJ and the adaptive threshold were used to count the number of bits from captured NIR images at different time points; these measurements were performed on five patches per group. For the signal intensity analysis, ImageJ and the adaptive threshold were used to evaluate the maximum pixel value in each NIR bit in captured images at different time points; these measurements were performed on 50–96 bits per patch for three patches per group. To evaluate the signal intensities between different time points, a consistent gain and exposure combination was used for fair comparisons.

### RM ECC for 2D binary array generation

Information bits of interest were encoded with redundancy using a RM ECC. The Python package ‘reedmuller’ (https://pypi.org/project/reedmuller/) was used for RM error correction encoding and decoding. The encoded binary string was then mapped to a 2D binary array after referring to a 2D template for orientation. The RM code with parameters *r* and *m*, denoted the RM(*r*, *m*) code, encodes $$k={\sum }_{i=0}^{r}\left(\begin{array}{c}m\\ i\end{array}\right)$$ information bits with 2^*m*^ string bits and is capable of correcting *c* = (2^*m*–*r*–1^ – 1) independent error bits. There is a trade-off between the number of information bits *k* and the maximum correctable error bits *c*, depending on the order *r*. For a 10 × 10 MNP with a RM(1, 6) code, the number of available string bits is ${2}^{\left\lfloor {\log }_{2}(10\times 10)\right\rfloor }=64$, where ⌊⌋ is the floor function, which results in encoding 7 information bits into a 64-bit string. The rest, 36 bits, were reserved for orientation correction. Nine orientation bits (3 × 3 size) were allocated to four corners of the 2D array (three corners ON and the bottom right corner OFF) for patch orientation and registration of rotation angles. Encoded binary string bits were arranged sequentially from top left to bottom right (omitting orientation bits at the four corners) in a raster scan manner. For a 12 × 12 MNP with *r* = 2, the RM(2, 7) code can encode 526.8 million different information combinations and guarantee to correct up to 15 error bits. For a 17 × 17 MNP with *r* = 2, the RM(2, 8) code can encode 137.4 billion different information combinations and guarantee to correct 31 error bits. This means that the desired error correction capability can be predetermined and one can select a RM(*r*, *m*) option to fit the use case. With a RM ECC, the proposed OPMR technology can generate 137.4 billion different patterns with 1.7 cm × 1.7 cm MNPs, which can accommodate the large number of varieties of vaccination information, the eight billion people in the human population on Earth or the fast-growing number of therapeutics that are available or currently under development.

### Encryption mask for patient data privacy

The OPMR system provides a high level of medical privacy, achieved through the strategic implementation of advanced security-preserving technologies across three layers. First, we use NIR fluorescent QDs that require a specific excitation wavelength (short-wavelength NIR at 780 nm) and a high-pass or band-pass filter mounted on the phone to receive only the emitted light (at the peak of 890–897 nm). Second, similar to password-protected hardware or software, we have a specific encryption procedure implemented in our OPMR system. Adding a known and fixed encryption pattern ensures the privacy of the personal medical data in the OPMR system. To decipher the encoded information, one must know not only the forward encoding method, but also the decipher key (that is, the fixed encryption mask). An encryption mask, composed of roughly half ON bits and half OFF bits, was added to the initially generated 2D pattern in a pixel-wise manner, applying a logical exclusive operator or an XOR operator on the initial encoded pattern. If an ON bit existed on the same pixel coordinate of both the encoded pattern and mask pattern, the pixel value was flipped. This step made the number of ON and OFF pixels even on the MNP, which made the recognition system robust to any pattern during the decoding step, because a RM code is highly structured and can have limited pixels in certain region or rows/columns. After randomly flipping pixels on the raw encoded pattern, the encrypted pattern will consist of half ON and half OFF pixels on average, which makes the recognition system robust to any patterns during the decoding step.

### Synthetic image generation for deep learning network training

Simulated fluorescence images were constructed for training deep learning networks. The synthetic training set included a variety of patterns, NIR bit spacings, locations, intensities, background noise levels and contrasts, MNP scales, rotations (from –5° to 5°), distortions, image qualities, defocusing levels, motion blurs, brightnesses, exposures, gains and more to consider potential image formation variations at three levels: the formation of a MNP, the formation of an image and the acquisition of a fluorescence image. To capture the temporal variations, the percentage of ON pixels was varied by 100%, 75%, 50% and 25%, for the 5%, 15%, 60% and 20% portions of the image dataset, respectively. A deep learning-assisted rectification network was applied to these synthetic models to output paired results. Detailed parameter settings and code for synthetic image generation can be found at https://github.com/liuyang12/ecc-microneedle. These paired results were input (90% for training and 10% for validation) to a convolutional-neural-network-based recognition network for it to learn the mapping of the input images to binary arrays. We used paired data to train models for 10 × 10 and 17 × 17 MNPs separately, each with 650,000 images (90% for training and 10% for validation). This large number in the synthetic training set improved the robustness of the recognition system overall. For all network training and analysis, Microsoft Excel v.2021, GraphPad Prism v.10, FIJI v.2017, Python v.3.8, PyTorch v.1.11 and MATLAB v.R2023b were used.

### Deep learning network for image binarization

The image binarization network was directly adapted from an off-the-shelf convolution network, U-Net, which was originally proposed for biomedical image segmentation. The binarization network took a single-channel (greyscale) 256 × 256 image as an input and output a two-channel 256 × 256 mask for final binarization. We used the cross-entropy loss function as the training criterion. During training, we used the same data generation process as the recognition network. This was an image-based ConvNet, which is light and accurate and easier to train with a given amount of training examples. To address binarization issues caused by impulse noise, we used 250,000 additional images with impulse noise augmentation (that is, salt and pepper noise, random erasing small rectangles and random Gaussian blur).

### Minimum area rectangle

As the second rectification step, a rectangle with the minimum area that covers all the white bits of the 2D array region was generated using an off-the-shelf Python implementation by OpenCV, ‘cv.minAreaRect()’. The selected region was then rotated, cropped and resized to a target size. The final crop size was 35% larger than the size of the minimum area rectangle while preserving the centre of the rectangle as a reference point. This rectification step was essential for efficient network training of the recognition model because keeping 2D arrays centred and normalized to the same scale reduces the amount of spatial variety (that is, data augmentation for training).

### Convolutional neural network for image recognition

We used a convolutional-neural-network-based network structure for the recognition model. We used the same network whether the MNP was 10 × 10 or 12 × 12. The input image sizes were 120 × 120 and 136 × 136, respectively, and these two models were trained separately using training samples with corresponding sizes. The input single-channel image was convolved by a 3 × 3 kernel with zero padding (adding zeros to the boundaries to keep the same image size) and then down-sampled to half the size. The same 3 × 3 convolution and down-sampling were repeated two times. The second convolution layer used no padding to obtain the target binary array size. After three convolution plus down-sampling layers, the 128-channel tensor was convolved by a 5 × 5 kernel without padding and finally convolved by a 1 × 1 kernel to get a two-channel mask for the final binary array. We used the cross-entropy loss function as the training criterion. BinarizationNet required the MNP size (*N* × *N*) as the input, while it was independent of the MNP size. The current OPMR system uses a laptop to run Python-based image processing and pattern recognition codes. In the future, this fully parameter-free, end-to-end structure could be converted to a Java-based smartphone application and combined with our image acquisition device to make it a stand-alone mobile system. This plug-and-play module could pave the way for easy deployment of the OPMR. These advancements would bring the OPMR technology one step closer to being clinically translational and readily applicable in the field. For all network training and analysis, Microsoft Excel v.2021, GraphPad Prism v.10, FIJI v.2017, Python v.3.8, PyTorch v.1.11 and MATLAB v.R2023b were used.

### Histological evaluation of OPMR MNP applications in swine

Pig skin tissues were excised after various time points post OPMR MNP application for histological semi-quantitative evaluations of the effect of the dye in the skin over time. The skin sections were evaluated by a board-certified veterinary pathologist (S.E.C.). Sections were examined and photographed using an Olympus BX45 microscope attached to a DP26 digital camera (Olympus). Skin lesions were graded for epidermal hyperplasia, hyperkeratosis, neutrophilic and eosinophilic inflammation, mononuclear leucocytic inflammation and dermal fibrosis. Neutrophilic/eosinophilic inflammation, mononuclear inflammation and hyperkeratotic lesions were graded with a numerical score from 0 to 4, in which 0 = normal, 1 = minimal, 2 = mild, 3 = moderate and 4 = severe. Epidermal hyperplasia and dermal fibrosis were graded from 0 to 3, in which 0 = normal, 1 = mild, 2 = moderate and 3 = severe. The scores for each parameter were averaged from 6–12 skin sections from 3–6 patches per group from 2–3 pigs. Specific histopathology scores between experimental groups or between time points were compared by a Mann Whitney *U*-test.

Additionally, quantification of cleaved caspase-3 (CC3) staining of pig tissue with no treatment, and with MNP applications loaded with just PVA/PVP polymer, blank PMMA microparticles or QD–PMMA microparticles were applied three days prior to skin excision. To study if the OPMR system activates cell apoptotic mechanisms, tissue samples where the OPMR dye was deposited were stained with CC3, which is a marker used to detect changes in cellular morphology (for example, shrinkage and degeneration, nuclear condensation and fragmentation) and which is particularly useful to detect apoptotic cells. The 2D image analysis of ten tissue cross-sections from each group tested and stained with CC3 was performed using the open-source software QuPath and ImageJ. An area approximately 2 mm in depth and 8 mm in length across the epidermis surface was manually defined for all images. Preprocessing steps were applied on each image to prepare them for subsequent analysis using an image analysis thresholder. The thresholder was created to isolate tissue samples from the image background and designate these sections as regions of interest in the software. First a colour transform on the image was applied, which provided a clear and binary way to contrast between positive (brown) and negative (purple) stained areas in the tissue. Then, the regions of interest were exported to ImageJ. In the exported binary image, the threshold of intensity indicating the positive staining was set. This threshold was set by manually checking, with trial and error, which threshold resulted in a more accurate depiction of positive versus negative staining. Using the threshold function of ImageJ, we were able to find the percentage of tissue area that clears that threshold, which corresponds to CC3-positive regions. Quantitative analysis of CC3 staining showed no differences in the apoptotic cell percentage between the control and experimental groups, indicating there was no signal of immunoreactivity in the skin sections.

### OPMR dye cytotoxicity analysis

For the OPMR dye (QD nanoparticles encapsulated in PMMA) cytotoxicity analysis^51^, HeLa cells were cultured in high glucose Dulbecco’s Modified Eagles Medium with phenol red (DMEM, Invitrogen) supplemented with 10% fetal bovine serum (Invitrogen) and 1% antibiotic (Invitrogen). Some 5,000 cells were seeded in a 96-well plate in full growth medium. Twenty hours after seeding, the media were replaced with fresh media including QD–PMMA microparticles at different concentrations and the cells were incubated for 20 h. Then the media were removed, cells were washed once with PBS buffer, MTS (Abcam) was added at 20% v/v in DMEM, cells were incubated for 4 h and absorbance was measured at 490 nm.

Additionally, human dermal fibroblasts were also cultured in fibroblast growth media (Invitrogen) supplemented with 1% penicillin/streptomycin (Invitrogen). Cells were seeded at a density of 5,000 cells per well in a 96-well plate containing full growth medium. Twenty hours post-seeding, the media were replaced with fresh media containing varying concentrations of QD–PMMA microparticles, alongside a fresh media control, and the cells were further incubated for 20 h. To assess cell viability, the live–dead assay and Cell Counting Kit-8 (CCK-8) were conducted. For the live–dead assay, cell media were aspirated, and the cells were gently washed once with PBS buffer. Cell viability was evaluated using the LIVE/DEAD Viability/Cytotoxicity Kit (Invitrogen, L3224) according to the manufacturer’s protocol and imaged under a DeltaVision Ultra microscope. The ratio of live cells to total cells was quantified as the ratio of viable cells to total cells using ImageJ. For the CCK-8 assay (Sigma-Aldrich), following media removal and PBS washing, cells were incubated with CCK-8 solution for 4 h and absorbance was measured at 450 nm. The viable cell number from each experimental group was normalized to the untreated control.

### The mRNA-LNP concentration and encapsulation efficiency analyses

The mRNA concentration and encapsulation efficiency in the LNPs was quantified using a Quant-iT RiboGreen assay (Thermo Fisher) and a modified procedure described elsewhere^43^. The encapsulated mRNA in LNPs were evaluated by quantifying the difference of mRNA concentrations in ×1 Tris–EDTA buffer and in 4% Triton X-100 buffer. The concentration of total mRNA was quantified by diluting mRNA-LNPs in Triton X-100 buffer. To quantify mRNA loading in MNPs by mass, microneedles were cut and dissolved in Tris–EDTA and Triton X-100. Subtracting the unencapsulated mRNA from the total mRNA yields the mRNA encapsulation efficiency.

### The mRNA and LNP quality assessments

The mRNA-LNPs were qualitatively assessed using cryo-TEM with and without QD–PMMA microparticles. Samples (0.1–0.5 mg ml^–1^) were added onto the carbon-coated copper TEM grids and the excess solution was blotted. Next, samples were plunge-frozen using a 930 Gatan Cryo-Plunge3 (Gatan). All the samples were imaged using a JEOL 2100 field-emission gun microscope (JEOL) at 200 kV acceleration voltage. Hydrodynamic size and polydispersity of mRNA-LNPs were analysed using DLS on a Zetasizer Nano-NS (Malvern Instruments) with and without QD–PMMA microparticles. Some 5 µl of the samples was diluted in 995 µl of UltraPure water in polystyrene cuvettes for size measurements. Three technical replicates were performed for each sample. The mRNA integrities in LNPs were assessed using a FEMTO pulse RNA fragment analyser (Agilent Technologies)^52^ with and without QD–PMMA microparticles. Some 2 μl of the samples with 0.5 pg μl^–1^ dilution in UltraPure water was loaded per lane. Three technical replicates were performed for each sample.

### SARS-CoV-2 vaccination with mRNA–OPMR MNPs in rats

Six-week-old to eight-week-old female Wistar rats were used for immunogenicity tests from vaccination via IM injection, mRNA MNP administration and mRNA–OPMR MNP administration. Each MNP contained 10 µg of encapsulated mRNA encoding SARS-CoV-2 S-protein RBD, and as a positive control, a matching dose of fresh mRNA-LNPs suspended in PBS was administered intramuscularly (*n* = 6 per group). MNPs were applied to the back area using an applicator for 20 min, and IM injections were administered to the thigh muscle. All animals received a booster dose via the same method (that is, IM injection, mRNA MNP or mRNA–OPMR MNP) at 28 days after the prime dose. Rats were bled at 3 and 7 weeks after the prime dose, and enzyme-linked immunosorbent assay (ELISA) was performed to evaluate anti-RBD binding titres.

### SARS-CoV-2 anti-RBD binding titres

Anti-RBD binding titres in rats were analysed using SARS-CoV-2 S-protein RBD ELISA detection. Recombinant SARS-CoV-2 S-protein RBD (1 μg ml^–1^, 100 μl per well, overnight at 4 °C; ACROBiosystems, SPN-C52H9) was used to capture anti-RBD IgG titres in rat serum (serial dilutions in PBS, 2 h at 37 °C). Goat polyclonal antibody (pAb) to rat IgG horseradish peroxidase conjugates (Abcam, ab112767) were used as secondary antibodies (1:10,000 dilution in blocking buffer, 100 μl per well, 1 h at 37 °C), and 3,5,3′,5′-tetramethylbenzidine (TMB) was used as a substrate (100 μl, 5 min incubation before addition of 100 μl of 3 N H_2_SO_4_). End-point titres were calculated as the dilution that emitted an optical density exceeding ×3 of the background produced by serum from naive mice.

### Pseudovirus-based neutralization assay

The SARS-CoV-2 pseudoviruses WA1/2020 strain (Wuhan/WIV04/2019, Global Initiative on Sharing All Influenza Data (GISAID) accession no. EPI_ISL_402124), expressing a luciferase reporter gene, were generated. In brief, the packaging plasmid psPAX2 (AIDS Resource and Reagent Program), luciferase reporter plasmid pLenti-CMV Puro-Luc (Addgene) and spike protein expressing pcDNA3.1 SARS-CoV-2 SΔCT of variants were co-transfected into HEK293T cells (ATCC, mycoplasma tested) using Lipofectamine 2000 (Thermo Fisher). The supernatants containing the pseudotype viruses were collected 48 h after transfection, and then were purified by centrifugation and filtration with a 0.45 µm filter. To determine the neutralization activity of the plasma or serum samples from participants, HEK293T-hACE2 cells were seeded in 96-well tissue culture plates at a density of 1.75 × 10^4^ cells per well overnight. Threefold serial dilutions of heat-inactivated serum or plasma samples were prepared and mixed with 50 µl pseudovirus. The mixture was incubated at 37 °C for 1 h before being added to HEK293T-hACE2 cells. Forty-eight hours after infection, cells were lysed in Steady-Glo Luciferase Assay (Promega) according to the manufacturer’s instructions. SARS-CoV-2 neutralization titres were defined as the sample dilution at which a 50% reduction in relative light unit was observed relative to the average of the virus control wells. The results were analysed using ordinary two-way ANOVA (Sidak’s multiple comparisons test).

### Firefly luciferase mRNA expression in rats

Six-week-old to eight-week-old female Wistar rats were used to test the delivery of mRNA encoding FLuc when administered with OPMR MNPs. MNPs loaded with 5 μg FLuc mRNA encapsulated in LNPs with or without OPMR dye in 17 × 17 arrays were fabricated and applied to the back area using a custom applicator with a velocity of 1,407 cm s^–1^ and holding force of 1.1 MPa for 10 min. Six hours after application, rats were imaged for bioluminescence of luciferase expression using an in vivo imaging system that was also a kinetic imaging system (PerkinElmer). Fifteen minutes prior to imaging, rats were injected with IVISbrite d-luciferin potassium salt XenoLight (PerkinElmer) intraperitoneally at 150 mg kg^–1^. Luminescence was quantified using LivingImage software (PerkinElmer).

### Statistical analysis

All in vitro and ex vivo experiments were performed in experimental triplicate or quintuplicate unless noted otherwise. All in vivo experiments were performed with five or six experimental replicates unless noted otherwise. Statistics analyses were performed using GraphPad Prism software using a two-tailed Student’s *t*-test for pairwise comparisons (non-statistical significance, *P* > 0.05). For multiple comparisons, one-way ANOVA was used unless noted otherwise. In all figures, data are presented as mean values, and ±s.d. is used for error bars.

### Ethics and inclusion declarations

Massachusetts Institute of Technology is dedicated to offering a safe, respectful, friendly and collegial environment for the benefit of everyone who attends, and for the advancement of the interests that bring us together. All animal procedures were approved and performed under the guidelines of the Massachusetts Institute of Technology Committee on Animal Care. Three-month-old female Yorkshire pigs were provided by Cummings School of Veterinary Medicine (protocol 0919-058-22). Six-week-old female Wistar rats were purchased from Charles River (protocol 0916-057-20).

### Reporting summary

Further information on research design is available in the Nature Portfolio Reporting Summary linked to this article.

## Online content

Any methods, additional references, Nature Portfolio reporting summaries, source data, extended data, supplementary information, acknowledgements, peer review information; details of author contributions and competing interests; and statements of data and code availability are available at 10.1038/s41563-024-02115-4.

## Supplementary information

Supplementary Information Competing interests of corresponding authors.

Reporting Summary

## Data Availability

All data generated or analysed during this study are included in the published Article and Extended Data figures and are available from the corresponding authors upon request.

## Code availability

Codes used for image binarization, image rectification and image recognition during this study are included in the published Article and Extended Data figures and are available via GitHub at https://github.com/liuyang12/ecc-microneedle.
