# Supplementary material for: On-patient medical record and mRNA therapeutics using intradermal microneedles
Source: Nat Mater. 2025 Feb 24;24(5):794–803. doi: 10.1038/s41563-024-02115-4 (PMC12048341; doi:10.1038/s41563-024-02115-4)
Supplement: Supplementary file 1 — Competing interests of corresponding authors. [file 41563_2024_2115_MOESM1_ESM.pdf]

nature materials

Supplementary information https://doi.org/10.1038/s41563-024-02115-4

On-patient medical record and mRNA therapeutics using intradermal microneedles

In the format provided by the authors and unedited

2024 Competing Interests Disclosure

From FY 2019 to the present, Dr. Robert Langer receives licensing fees (to patents in which he was an inventor on) from, invested in, consults (or was on Scientific Advisory Boards or Boards of Directors) for, lectured (and received a fee), or conducts sponsored research at MIT for which he was not paid for the following entities:

1. 611 Therapeutics
2. Abpro International;
3. Acorda (Formerly Civitas Therapeutics);
4. Alfred University;
5. Aleph Farms;
6. Alivio Therapeutics;
7. Alkermes;
8. Allevi;
9. Allurion;
10. Alnylam Pharmaceuticals, Inc;
11. Amberstone Bioscience;
12. Amgen;
13. aMoon
14. Apotex;
15. Arcadia Biosciences, Inc;
16. Arsenal Medical;
17. Artificial Cell Technology, Inc;
18. Avalon-Globocare;
19. Bai Biosciences;
20. BASF Corporation;
21. Bayer;
22. Balzan Foundation;
23. Bexson Biomedical
24. Bilayer Therapeutics;
25. Biogen;
26. BioInnovation Institute (Novo Nordisk Founden);
27. BioTE Medical;
28. Blackrock;
29. Blackstone (Formerly Clarus);
30. Boston Children's Hospital;
31. CBC Group Investment Mgmt Group;
32. Celanese;
33. Celero;
34. Cellink/BICO
35. Cellomics Technology, Llc;
36. Cellular Biomedical;
37. CE&N/ ACS
38. Charles River Laboratories, Inc.;
39. ClavystBio
40. Clontech Laboratories;
41. Combined Therapeutics ("CTx");
42. Conference Forum;
43. Coregen
44. Cornell University;
45. Crispr Therapeutics Ag;
46. Crown Bioscience Inc.;
47. CyMon Bio
48. Daré Biosciences (Formerly Microchips Biotech, Juniper Pharmaceuticals, and Columbia Laboratories);
49. Daros, Inc.
50. DeepBiome;
51. Dewpoint Therapeutics;
52. Dispendix;
53. Eagle Pharmaceuticals;
54. Earli;
55. Edigene Biotechnology, Inc.;
56. Editas Medicine, Inc.;
57. ELC (Estee Lauder Companies);
58. Eli Lilly;
59. Eisai Inc.;
60. Entrega;
61. EpiBone
62. Establishment Labs, SA.
63. Everlywell;
64. Evox Therapeutics, Ltd.;
65. Fate
66. Flagship Pioneering;
67. Frequency Therapeutics, Inc.;

2024 Competing Interests Disclosure

From FY 2019 to the present, Dr. Robert Langer receives licensing fees (to patents in which he was an inventor on) from, invested in, consults (or was on Scientific Advisory Boards or Boards of Directors) for, lectured (and received a fee), or conducts sponsored research at MIT for which he was not paid for the following entities:

68. GeneLeap Biotech
69. Genemedicine Co Lmtd;
70. GenScript USA Inc;
71. Geneo Medicine;
72. GENUV;
73. Glaxosmithkline Llc;
74. Glycobia;
75. Glympse Bio;
76. Goldman Sachs
77. Greenlight Biosciences;
78. HERVOLUTION Therapeutics
79. HCR (HealthCare Royalty Partners);
80. HKF DNA Technologies;
81. Hopewell Therapeutics;
82. Horizon Discovery Group Plc;
83. Humacyte, Inc.;
84. IBEX Pharmaceuticals, Inc.;
85. Immunai;
86. ImmuneXcite Inc.;
87. Institute of Immunology Co. Ltd;
88. Integrated DNA Technologies, Inc.;
89. InVivo Therapeutics;
90. IxBio;
91. Jimini Health, Inc.
92. J.R. Simplot Company;
93. Jnana Therapeutics;
94. Kala Pharmaceuticals;
95. Kallyope, Inc.;
96. Kendall Capital;
97. Kensa;
98. Kodikaz Therapeutics;
99. KAST (Korean Academy of Science and Technology);
100. Ksq Therapeutics, Inc.;
101. Kunlun Capital;
102. Landsdowne Labs;
103. LikeMinds;
104. Lindus Health
105. Lonza;
106. Luminopia, Inc.;
107. Luye (Shandong luye);
108. Lyndra Therapeutics;
109. Lyra Therapeutics (Formerly "480 Biomedical");
110. Marble Therapeutics;
111. Maurice Marie Janot Award 2020
112. McGovern Institute;
113. Medikinetics Co., Ltd.;
114. Merck;
115. MGH Ragon Institute;
116. Micelle;
117. Moderna Therapeutics;
118. Momenta;
119. Muse Biotechnologies Inc.
120. Mylan;
121. N2Tech;
122. Nanobiosym;
123. Nanobiotix;
124. Neochromosone;
125. Neoteny 4 LLP;
126. NextRNA;
127. Newbridge Ventures LLC;
128. Noveome Biotherapeutics, Inc.;
129. Novo Nordisk;
130. Ohio State University;
131. Olivo (acquired by Shiseido)
132. OmniPulse
133. Ovid Therapeutics;
134. Particles for Humanity;
135. Pfizer, Inc.;

2024 Competing Interests Disclosure

From FY 2019 to the present, Dr. Robert Langer receives licensing fees (to patents in which he was an inventor on) from, invested in, consults (or was on Scientific Advisory Boards or Boards of Directors) for, lectured (and received a fee), or conducts sponsored research at MIT for which he was not paid for the following entities:

136. Pioneer Hi-Bred International, Inc.;
137. Placon Therapeutics
138. Polaris Partners;
139. Pontifical Academy of Sciences;
140. Portal Instruments;
141. Preceres, Llc (Acquired by Monsanto);
142. PrognomIQ Inc.;
143. Pulmatrix;
144. PureTech;
145. Quris
146. ReLive;
147. Rensselaer Polytechnic Institute/ Department of Chemical and Biological Engineering
148. Reprocell Usa, Inc. (Formerly Stemgent);
149. Replay Bio;
150. Rubius Therapeutics;
151. Satellite Bio;
152. SBEF (Seoul Bio Economy Forum);
153. Secant Medical, Inc.;
154. Seer, Inc.;
155. Selecta Biosciences;
156. Senses LLC;
157. Setsuro Tech Inc.;
158. Seventh Sense Biosystems, Inc.;
159. Shenzhen Rice Life Technology, Ltd;
160. Shire Ag;
161. Sigilon;
162. Sigma Aldrich Co. Llc;
163. Sio2;
164. Ske S.R.L.;
165. Soil Culture Solutions Llc (Dba Soilcea);
166. Souffle Therapeutics;
167. Solvandria Foundation
168. SQZ Biotechnologies;
169. StemBioSys, Inc.;
170. SuonoBio;
171. Syntis Bio, Inc;
172. T2 Biosystems;
173. Taconic Biosciences, Inc. (formerly Taconic Farms);
174. Taiwania Capital (Bio-Asia Taiwan Symposium);
175. TARA;
176. Tarveda Therapeutics;
177. Teal Bio
178. Terasaki Institute
179. Tesio Pharmaceuticals
180. Third Rock Ventures;
181. Tiba Biotech LLC;
182. TissiuM (formerly"Gecko");
183. Transgenic Inc.;
184. Translate Bio (Formerly Rana Therapeutics, Inc.);
185. Trilink Biotechnologies, Inc.;
186. T.Rx Capital, LLC
187. Unilever (Living Proof);
188. University of Bergen, Norway (Falch Lecture Honorarium);
189. VasoRX;
190. Verseau Therapeutics, Inc.;
191. Virex Health
192. Vitakey;
193. Vivtex Corporation;
194. Westlake University;
195. Whitehead Institute;
196. Wiki Foods;
197. Xenter;
198. YourBio (Formerly 7th Sense Biosystems)
199. Yz Biosciences (Guangzhou) Inc.;
200. Zenomics;
201. ZWI Therapeutics

2024 Competing Interests Disclosure

From FY 2019 to the present, Dr. Ana Jaklenec receives licensing fees (to patents in which she was an inventor on) from, invested in, consults (or was on Scientific Advisory Boards or Boards of Directors) for, lectured (and received a fee), or conducts sponsored research at MIT for which she was not paid for the following entities:

1. The Estée Lauder Companies
2. Moderna Therapeutics
3. OmniPulse Biosciences
4. Particles for Humanity
5. SiO2 Materials Science
6. VitaKey
