# Supplementary material for: On-patient medical record and mRNA therapeutics using intradermal microneedles
Source: Nat Mater. 2025 Feb 24;24(5):794–803. doi: 10.1038/s41563-024-02115-4 (PMC12048341; doi:10.1038/s41563-024-02115-4)
Supplement: Supplementary file 2 — Reporting Summary [file 41563_2024_2115_MOESM2_ESM.pdf]

nature portfolio

Corresponding author(s): Robert Langer, Ana Jaklenec

Last updated by author(s): November 07, 2024

Reporting Summary

Nature Portfolio wishes to improve the reproducibility of the work that we publish. This form provides structure for consistency and transparency in reporting. For further information on Nature Portfolio policies, see our Editorial Policies and the Editorial Policy Checklist.

Please do not complete any field with "not applicable" or n/a. Refer to the help text for what text to use if an item is not relevant to your study.
For final submission: please carefully check your responses for accuracy; you will not be able to make changes later.

Statistics

For all statistical analyses, confirm that the following items are present in the figure legend, table legend, main text, or Methods section.

n/a	Confirmed	
☐	☑	The exact sample size (n) for each experimental group/condition, given as a discrete number and unit of measurement
☐	☑	A statement on whether measurements were taken from distinct samples or whether the same sample was measured repeatedly
☐	☑	The statistical test(s) used AND whether they are one- or two-sided<br>*Only common tests should be described solely by name; describe more complex techniques in the Methods section.*
☐	☑	A description of all covariates tested
☐	☑	A description of any assumptions or corrections, such as tests of normality and adjustment for multiple comparisons
☐	☑	A full description of the statistical parameters including central tendency (e.g. means) or other basic estimates (e.g. regression coefficient) AND variation (e.g. standard deviation) or associated estimates of uncertainty (e.g. confidence intervals)
☐	☑	For null hypothesis testing, the test statistic (e.g. F, t, r) with confidence intervals, effect sizes, degrees of freedom and P value noted<br>*Give P values as exact values whenever suitable.*
☐	☑	For Bayesian analysis, information on the choice of priors and Markov chain Monte Carlo settings
☐	☑	For hierarchical and complex designs, identification of the appropriate level for tests and full reporting of outcomes
☐	☑	Estimates of effect sizes (e.g. Cohen's d, Pearson's r), indicating how they were calculated

Our web collection on statistics for biologists contains articles on many of the points above.

Software and code

Policy information about availability of computer code

Data collection	IR Record v2
Data analysis	Microsoft Excel 2021, GraphPad Prism 10, Image J FIJI 2017, Python 3.8; PyTorch 1.11; MATLAB R2023b

For manuscripts utilizing custom algorithms or software that are central to the research but not yet described in published literature, software must be made available to editors and reviewers. We strongly encourage code deposition in a community repository (e.g. GitHub). See the Nature Portfolio guidelines for submitting code & software for further information.

Data

Policy information about availability of data

All manuscripts must include a data availability statement. This statement should provide the following information, where applicable:

- Accession codes, unique identifiers, or web links for publicly available datasets
- A description of any restrictions on data availability
- For clinical datasets or third party data, please ensure that the statement adheres to our policy

All data generated or analyzed during this study are included in the published article and Extended DataSupplementary Figures Information and are available from the corresponding authors upon reasonable request.

Research involving human participants, their data, or biological material

Policy information about studies with human participants or human data. See also policy information about sex, gender (identity/presentation), and sexual orientation and race, ethnicity and racism.

Reporting on sex and gender	N/A
Reporting on race, ethnicity, or other socially relevant groupings	N/A
Population characteristics	N/A
Recruitment	N/A
Ethics oversight	N/A

Note that full information on the approval of the study protocol must also be provided in the manuscript.

Field-specific reporting

Please select the one below that is the best fit for your research. If you are not sure, read the appropriate sections before making your selection.

☑ Life sciences ☐ Behavioural & social sciences ☐ Ecological, evolutionary & environmental sciences

For a reference copy of the document with all sections, see nature.com/documents/nr-reporting-summary-flat.pdf

Life sciences study design

All studies must disclose on these points even when the disclosure is negative.

Sample size	All in vitro and ex vivo experiments were performed in experimental triplicate or quintuplicate unless noted otherwise. 6 tissue samples per biological replicate were used unless noted otherwise. All in vivo experiments were performed with five or six experimental replicates unless noted otherwise. These numbers were selected as they exhibited good consistency and repeatability.
Data exclusions	No data was excluded if a microneedle patch was appropriately applied.
Replication	All in vitro and ex vivo experiments were performed in experimental triplicate or quintuplicate unless noted otherwise. 6 tissue samples per biological replicate were used unless noted otherwise. All in vivo experiments were performed with five or six experimental replicates unless noted otherwise. These exhibited good consistency and repeatability.
Randomization	Animals were randomized into experimental groups based on a random numbering system assigned upon arrival. All non-animal studies (e.g., in vitro, ex vivo) were randomized by randomly selecting tissue samples upon application.
Blinding	No blinding was performed. OPMR studies are mainly longitudinal, therefore, we must track individual animals for long-term.

Behavioural & social sciences study design

All studies must disclose on these points even when the disclosure is negative.

Study description	N/A
Research sample	N/A
Sampling strategy	N/A
Data collection	N/A
Timing	N/A
Data exclusions	N/A
Non-participation	N/A
Randomization	N/A

Ecological, evolutionary & environmental sciences study design

All studies must disclose on these points even when the disclosure is negative.

Study description	N/A
Research sample	N/A
Sampling strategy	N/A
Data collection	N/A
Timing and spatial scale	N/A
Data exclusions	N/A
Reproducibility	N/A
Randomization	N/A
Blinding	N/A

Did the study involve field work? ☐ Yes ☑ No

Field work, collection and transport

Field conditions	N/A
Location	N/A
Access & import/export	N/A
Disturbance	N/A

Reporting for specific materials, systems and methods

We require information from authors about some types of materials, experimental systems and methods used in many studies. Here, indicate whether each material, system or method listed is relevant to your study. If you are not sure if a list item applies to your research, read the appropriate section before selecting a response.

Materials & experimental systems

n/a	Involved in the study	
☐	☑	Antibodies
☐	☑	Eukaryotic cell lines
N/A	☐	Palaeontology and archaeology
☐	☑	Animals and other organisms
N/A	☐	Clinical data
N/A	☐	Dual use research of concern
N/A	☐	Plants

Methods

n/a	Involved in the study	
N/A	☐	ChIP-seq
N/A	☐	Flow cytometry
N/A	☐	MRI-based neuroimaging

Antibodies

Antibodies used	Recombinant SARS-CoV-2 S-protein RBD (ACROBiosystems, SPN-C52H9)<br>Goat pAb to rat IgG horseradish peroxidase conjugates (Abcam, ab112767)
Validation	All antibodies were validated using positive and negative control samples and were utilized according to the manufacturer's recommendations.

Eukaryotic cell lines

Policy information about cell lines and Sex and Gender in Research

Cell line source(s)	HeLa (Henrietta Lacks) cells and HEK293T-hACE2 cells (Human Embryonic Kidney) (ATCC, mycoplasma tested)
Authentication	All cell lines used in the study are commercial lines that have been authenticated by the supplier.
Mycoplasma contamination	These cell lines are not individually checked for Mycoplasma; but they are maintained in specialized incubators following the Contracon decontamination protocol to prevent Mycoplasma contamination.
Commonly misidentified lines (See ICLAC register)	N/A

Palaeontology and Archaeology

Specimen provenance	N/A
Specimen deposition	N/A
Dating methods	N/A

☐ Tick this box to confirm that the raw and calibrated dates are available in the paper or in Supplementary Information.

Ethics oversight	N/A

Note that full information on the approval of the study protocol must also be provided in the manuscript.

Animals and other research organisms

Policy information about studies involving animals; ARRIVE guidelines recommended for reporting animal research, and Sex and Gender in Research

Laboratory animals	All animal procedures were approved and performed under the guidelines of the Massachusetts Institute of Technology Committee on Animal Care. Three months old, female, Yorkshire pigs were provided by Cummings School of Veterinary Medicine (Grafton, MA) (Protocol 0919-058-22). Six weeks old, female, Wistar rats were purchased by Charles River (Cambridge, MA) (Protocol 0916-057-20).
Wild animals	No wild animals were used in this study.
Reporting on sex	Female Wistar rats and female swine.
Field-collected samples	No field collected samples were used in this study.
Ethics oversight	All animal procedures were approved and performed under the guidelines of the Massachusetts Institute of Technology Committee on Animal Care and were overseen by the Division of Comparative Medicine (DCM) in an AALAC accredited facility.

Note that full information on the approval of the study protocol must also be provided in the manuscript.

Clinical data

Policy information about clinical studies

All manuscripts should comply with the ICMJE guidelines for publication of clinical research and a completed CONSORT checklist must be included with all submissions.

Clinical trial registration	N/A
Study protocol	N/A
Data collection	N/A
Outcomes	N/A

Dual use research of concern

Policy information about dual use research of concern

Hazards

Could the accidental, deliberate or reckless misuse of agents or technologies generated in the work, or the application of information presented in the manuscript, pose a threat to:

No	Yes	
x	☐	Public health
x	☐	National security
x	☐	Crops and/or livestock
x	☐	Ecosystems
x	☐	Any other significant area

Experiments of concern

Does the work involve any of these experiments of concern:

No	Yes	
x	☐	Demonstrate how to render a vaccine ineffective
x	☐	Confer resistance to therapeutically useful antibiotics or antiviral agents
x	☐	Enhance the virulence of a pathogen or render a nonpathogen virulent
x	☐	Increase transmissibility of a pathogen
x	☐	Alter the host range of a pathogen
x	☐	Enable evasion of diagnostic/detection modalities
x	☐	Enable the weaponization of a biological agent or toxin
x	☐	Any other potentially harmful combination of experiments and agents

Plants

Seed stocks	N/A
Novel plant genotypes	N/A
Authentication	N/A

ChIP-seq

Data deposition

N/A Confirm that both raw and final processed data have been deposited in a public database such as GEO.

N/A Confirm that you have deposited or provided access to graph files (e.g. BED files) for the called peaks.

Data access links<br>*May remain private before publication.*	N/A
Files in database submission	N/A
Genome browser session<br>(e.g. UCSC)	N/A

Methodology

Replicates	N/A
Sequencing depth	N/A
Antibodies	N/A
Peak calling parameters	N/A
Data quality	N/A

Software	N/A

Flow Cytometry

Plots

Confirm that:

N/A The axis labels state the marker and fluorochrome used (e.g. CD4-FITC).

N/A The axis scales are clearly visible. Include numbers along axes only for bottom left plot of group (a 'group' is an analysis of identical markers).

N/A All plots are contour plots with outliers or pseudocolor plots.

N/A A numerical value for number of cells or percentage (with statistics) is provided.

Methodology

Sample preparation	N/A
Instrument	N/A
Software	N/A
Cell population abundance	N/A
Gating strategy	N/A

N/A Tick this box to confirm that a figure exemplifying the gating strategy is provided in the Supplementary Information.

Magnetic resonance imaging

Experimental design

Design type	N/A
Design specifications	N/A
Behavioral performance measures	N/A

Imaging type(s)	N/A
Field strength	N/A
Sequence & imaging parameters	N/A
Area of acquisition	N/A

Diffusion MRI ☐ Used ☑ Not used

Preprocessing

Preprocessing software	N/A
Normalization	N/A
Normalization template	N/A
Noise and artifact removal	N/A
Volume censoring	N/A

Statistical modeling & inference

Model type and settings	N/A
Effect(s) tested	N/A

Specify type of analysis: ☐ Whole brain ☐ ROI-based ☐ Both

Statistic type for inference (See Eklund et al. 2016)	N/A
Correction	N/A

Models & analysis

n/a	Involved in the study
N/A	☐ Functional and/or effective connectivity
N/A	☐ Graph analysis
N/A	☐ Multivariate modeling or predictive analysis

Functional and/or effective connectivity	N/A
Graph analysis	N/A
Multivariate modeling and predictive analysis	N/A

This checklist template is licensed under a Creative Commons Attribution 4.0 International License, which permits use, sharing, adaptation, distribution and reproduction in any medium or format, as long as you give appropriate credit to the original author(s) and the source, provide a link to the Creative Commons license, and indicate if changes were made. The images or other third party material in this article are included in the article's Creative Commons license, unless indicated otherwise in a credit line to the material. If material is not included in the article's Creative Commons license and your intended use is not permitted by statutory regulation or exceeds the permitted use, you will need to obtain permission directly from the copyright holder. To view a copy of this license, visit http://creativecommons.org/licenses/by/4.0/
